# Esophageal epithelial cell-derived kynurenine drives Th17 inflammation in reflux esophagitis and is targeted by Xuanfu Daizhe decoction

**DOI:** 10.1186/s13020-026-01413-0

**Published:** 2026-07-15

**Authors:** Jing Xun, Qi Gao, Huichao Yang, Jinlu Zhang, Yu Wang, Bin Liu, Xiaolin Jiang, Dihua Li, Lin Lang, Haiwei Mei, Ximo Wang, Qi Zhang, Yu Wu

**Affiliations:** 1 Tianjin Nankai Hospital, Tianjin Medical University, Tianjin, 300100 China; 2 Institute of Integrative Medicine for Acute Abdominal Diseases, Tianjin, 300100 China; 3 Tianjin Key Laboratory of Acute Abdomen Disease Associated Organ Injury and ITCWM Repair, Tianjin, 300100 China; 4 Tianjin Medical University, Tianjin, 300100 China

**Keywords:** Reflux esophagitis, Th17 cells, Kynurenine, AhR signaling pathway, Xuanfu Daizhe decoction

## Abstract

**Background:**

The role of immunometabolic dysregulation in reflux esophagitis (RE), particularly the metabolic drivers of pathogenic T helper 17 (Th17) cell differentiation, is not fully understood. Although Xuanfu Daizhe Decoction (XDD) is a clinically effective Traditional Chinese Medicine (TCM) formula for RE, its potential to modulate these immunometabolic pathways remains unelucidated.

**Objective:**

This study aimed to investigate the mechanism by which tryptophan metabolic dysregulation in RE influences Th17 cell differentiation and to evaluate whether XDD exerts therapeutic effects by targeting this pathway.

**Methods:**

Metabolomic analysis was performed to assess alterations in the tryptophan metabolic pathway in serum from RE patients and RE rat models. Inflamed human esophageal epithelial cells (HEECs) model was established using acidified bile salts to observe kynurenine (KYN) secretion and its effect on Th17 differentiation. siRNA-mediated silencing of the aryl hydrocarbon receptor repressor (AHRR) was employed to clarify the role of AHRR in KYN-AhR-mediated Th17 differentiation. The interventional effects of XDD and its impact on the KYN-AHRR-AhR-Th17 axis were further examined in both cellular and rat models.

**Results:**

Metabolomic profiling of clinical samples and RE rat models demonstrated significant dysregulation in the KYN pathway. Furthermore, we identified that inflamed HEECs induced high expression of IDO/TDO to elevate KYN secretion, which promoted Th17 cell differentiation. Mechanistically, KYN disrupted aryl hydrocarbon receptor (AhR) signaling via upregulation of its repressor, AHRR, thereby driving Th17 differentiation. Finally, we demonstrated that XDD treatment alleviated inflammation and tissue injury of RE by modulating the KYN-AHRR-AhR-Th17 axis.

**Conclusion:**

This study reveals a novel mechanism in RE whereby esophageal epithelial-derived KYN promotes Th17 differentiation via the AHRR–AhR signaling axis. Furthermore, XDD exerts therapeutic effects by modulating this immunometabolic pathway. These findings provide new insights into RE pathogenesis and experimental evidence supporting the application of XDD in immunometabolic disorders.

**Supplementary Information:**

The online version contains supplementary material available at 10.1186/s13020-026-01413-0.

## Introduction

Reflux esophagitis (RE) is a prevalent inflammatory disorder primarily caused by the retrograde flow of gastric contents into the esophagus [1]. Clinically, RE is characterize by chronic inflammation, mucosal damage [2], and alterations in immune [3] and metabolic responses [4], which may lead to complications such as dysphagia and, in severe cases, esophageal cancer*,* while the central role of immune dysregulation in RE pathogenesis is well-recognized, the precise mechanisms driving the persistent inflammatory response remain a focus of investigation.

Notably, T-helper 17 (Th17) cells—a pivotal pro-inflammatory T-helper subset cells—have emerged as pivotal contributors to esophageal tissue damage [5]. The signature cytokine, interleukin-17A (IL-17A), along with a range of other pro-inflammatory cytokines, amplifies tissue-destructive immunity [6]. It suggests the modulation of Th17 cell activity as a promising therapeutic strategy. However, the upstream factors that orchestrate Th17 differentiation and activation in the specific context of RE are not fully defined.

Recent evidence points to metabolic reprogramming as a critical regulator of immune cell function. In particular, the tryptophan-kynurenine metabolic axis, governed by the rate-limiting enzyme indoleamine 2,3-dioxygenase 1 (IDO1), has been implicated in shaping Th17 responses [7, 8]. The key metabolite, kynurenine, serves as an endogenous ligand for the aryl hydrocarbon receptor (AhR) [9, 10], a transcription factor that regulates the expression of retinoic acid-related orphan receptor gamma t (ROR-γt) and forkhead box P3 (Foxp3), two key transcription factors that directly influence the balance between pro-inflammatory Th17 and regulatory T (Treg) cells [11]. Kynurenine-dependent immune tolerance and inflammatory responses are therefore largely mediated by AhR signaling [12, 13]. Although the IDO1–kynurenine–AhR axis has been extensively studied in oncology and other immune-related disorders, its specific role in the pathogenesis of RE has not yet been clarified.

In the regulation of AhR signaling, the aryl hydrocarbon receptor repressor (AHRR) plays a critical role as a negative regulator [14]. AHRR inhibits AhR activation by competing with AhR for binding to ligands, thereby reducing AhR-mediated transcriptional activity. Recent studies have indicated that AHRR is involved in modulating immune responses and inflammation in various diseases [15]. It suggests that AHRR is implicated in promoting Th17-driven inflammation in RE, potentially through its inactivation of AhR.

Given the complexity of this immunometabolic crosstalk, interventions capable of simultaneously targeting multiple pathways hold significant promise. Traditional Chinese Medicine (TCM), a long history of treating complex inflammatory diseases, offers a unique approach to modulating immune and metabolic pathways [16]. RE belongs to the categories of “acid regurgitation”, “noisy stomach” and “esophageal inflammation” in TCM. Its core pathogenesis is “deficiency of the spleen and stomach, disorder of ascending and descending qi, adverse rising of stomach qi, and turbid pathogenic factors invading the esophagus”. Xuanfu Daizhe Decoction (XDD), originated from “Shang Han Lun”, is a classical TCM formula with the core efficacy of replenishing qi, harmonizing the stomach, descending counterflow and resolving phlegm, which is highly consistent with the core pathogenesis of RE. Multiple clinical studies have demonstrated that XDD effectively alleviates the cardinal symptoms of RE [17, 18]. Modern pharmacological studies have confirmed that XDD inhibits esophageal inflammation by regulating multiple signaling pathways, including the STAT1/TREM-1 pathway, PI3K/AKT signaling pathway, and NLRP3 inflammasome [19–21]. However, the underlying mechanisms by which XDD exerts its therapeutic effects in RE, particularly its interaction with the kynurenine-AhR-Th17 axis, remains to be elucidated.

In this study, we found that Th17/IL-17A levels correlate with disease severity in clinical RE patients, and identify dysregulated tryptophan metabolites in RE patient and a rat RE model via metabolomic analysis. We further demonstrated that inflammatory esophageal epithelial cells-derived kynurenine promotes Th17 differentiation and activation via AHRR-mediated inactivation of AhR. Finally, we determined that XDD exerted its effect in treating RE by regulating the kynurenine-AhR-Th17 axis and reducing inflammation in the esophageal epithelium cells. Our work will provide a novel mechanistic understanding of RE pathogenesis and establish a scientific foundation for XDD as a promising immunometabolic therapy.

## Materials and methods

### Subject selection

This study enrolled patients diagnosed with RE and healthy controls (HCs) at Tianjin Nankai Hospital, affiliated with Tianjin Medical University. Participants were aged between 35 and 84 years (Table 1). The diagnosis of RE was established based on endoscopic findings, which were evaluated according to the Los Angeles (LA) classification, following the specific criteria outlined in the “Lyon Consensus 2.0” [22]. Healthy controls were recruited from the same hospital. Exclusion criteria for all participants included: (1) use of proton pump inhibitors or gastrointestinal motility agents within one week prior to enrollment; (2) structural abnormalities of the digestive tract; (3) eosinophilic esophagitis; (4) Helicobacter pylori infection; (5) metabolic disorders; (6) coagulation dysfunction; (7) functional diseases affecting the heart, liver, kidneys, or other major organs; (8) hematological or immune system disorders; and (9) use of antibiotics, probiotics, or prebiotics within the two months prior to sample collection. Demographic data, including age, sex, and body mass index (BMI), along with gastrointestinal symptoms, routine blood test results, gastroscopy findings, and 24-h esophageal pH monitoring results (for RE patients), were systematically collected. All participants had not used antibiotics, probiotics, or prebiotics in the two months preceding sample collection [4]. Serum samples were subsequently obtained for both non-targeted metabolomics analysis and targeted tryptophan metabolomics analysis. Following standardized sample collection protocols, fresh serum samples were drawn at Tianjin Nankai Hospital using sterile collection tubes. These samples were immediately mixed thoroughly, transported on ice, and stored at − 80 °C until further analysis. Clinical characteristics and demographic information of all participants were meticulously recorded at the time of sample collection. The study was conducted in compliance with the ethical principles outlined in the Declaration of Helsinki. The protocol received approval from the Ethics Committee of Tianjin Nankai Hospital (NKYY_YXKT__IRB_2023_063_01), and all participants provided written informed consent prior to enrollment.

**Table 1:** Comparison of clinical features between RE group and healthy control group

Variable	Healthy control group (*n* = 15)	RE group (*n* = 35)	*P*
Age media (range)	60 (30–80)	62 (35–84)	0.535
Male, *n* (%)	5 (33.33%)	16 (45.71%)	0.4163
Female, *n* (%)	10 (66.67%)	19 (54.29%)	
Time of medical history (month)	0 (0–0)	20 (1–360)	< 0.0001
Main symptom reported, *n* (%)			
Chronic gastritis	0 (0%)	19 (54.29%)	
Heartburn or chest pain	0 (0%)	29 (82.86%)	
Regurgitation	0 (0%)	32 (91.43%)	
Vomiting	0 (0%)	25 (71.83%)	
Respiratory symptoms	0 (0%)	12 (34.29%)	
Psychological, mental problems	0 (0.00%)	0 (0.00%)	

Time of medical history is presented as median (range)

### Preparation of Xuanfu Daizhe decoction compound formula

Xuanfu Daizhe Decoction (XDD) consists of seven traditional Chinese medicinal herbs in fixed proportions: 15 g of Flos Inulae (Inulae Flos, Flower, Shandong, 20240430), 5 g of Haematitum (Hematite, Mineral, Hebei, 20240320), 10 g of Radix Ginseng (Ginseng, Root, Jilin, 20240426), 15 g of Rhizoma Pinelliae Preparatum (Pinelliae Rhizoma, Root, Gansu, 20240310), 15 g of Radix Glycyrrhizae Preparata (Liquorice Root, Root, Neimeng, 20240413), 25 g of Rhizoma Zingiberis Recens (Zingiberis Rhizoma Recens, Root, Tianjin), and 10 g of Fructus Jujubae (Jujube, Fruit, Hebei, 20240201). All herbs were sourced from the pharmacy department of Tianjin Nankai Hospital and were authenticated by a certified pharmacognosist according to the Chinese Pharmacopoeia (2025 edition). The herbs were obtained from the same supplier to ensure batch-to-batch consistency.

The preparation of XDD followed traditional decoction procedures with standardization of key steps. Specifically, Haematitum was first boiled in 10 times its weight of distilled water for 30 min. Then, the remaining herbs were added, with Flos Inulae wrapped in sterile gauze before decoction. The mixture was boiled for another 30 min and then filtered. The herbal residue was reboiled with eight times the weight of water for 30 min. The two filtrates were combined and concentrated under reduced pressure to a final concentration of 1 g/mL, corresponding to 1 g of crude herb per mL of extract. The decoction was stored at 4 °C until use.

To ensure consistency, the preparation was conducted according to a standardized protocol with fixed ratios, defined boiling times, and concentration procedures. All decoctions were prepared fresh in batches under identical laboratory conditions, and preparation records were maintained. These measures ensured the reproducibility of the herbal intervention, which is essential for studies evaluating its effects on gut microbiota.

### Nontargeted metabolomics analysis

A total of 40 RE patients and 40 healthy controls were enrolled in the overall cohort. For metabolomic profiling, 15 healthy controls and 35 RE patients were randomly selected from the full cohort, based on experimental design and cost considerations. Nontargeted metabolomics analysis was performed using liquid chromatography-mass spectrometry (LC–MS) as previously described by Kui et al. [23]. Serum samples (100 µL) were transferred to Eppendorf tubes, and 400 µL of 80% methanol solution was added. The samples were vortexed thoroughly and incubated on ice for 5 min. After incubation, the samples were centrifuged at 15,00×*g* for 20 min at 4 °C to separate the supernatant. The supernatant was then diluted to achieve a final methanol concentration of 53%, followed by an additional centrifugation step before being collected for further analysis. For ultrahigh-performance liquid chromatography-tandem mass spectrometry (UHPLC–MS/MS) analysis, a Vanquish UHPLC system (Thermo Fisher, Germany) was used in conjunction with an Orbitrap Q Exactive™ HF mass spectrometer (Thermo Fisher, Germany). Chromatographic separation was achieved using a Hypersil Gold C18 column maintained at 40 °C, with a flow rate of 0.2 mL/min. The mobile phases were: for positive ion mode, mobile phase A was 0.1% formic acid, and mobile phase B was methanol; for negative ion mode, mobile phase A consisted of 5 mM ammonium acetate, and mobile phase B was methanol. The mass spectrometry parameters were optimized as follows: spray voltage at 3.5 kV, sheath gas flow rate at 35 psi, auxiliary gas flow rate at 10 L/min, ion transfer tube temperature at 320 °C, RF level for ion transfer at 60, and auxiliary gas heater temperature at 350 °C. Both positive and negative ion modes were used for analysis, with data-dependent MS/MS secondary scanning employed. Raw data were processed using CD 3.1 library search software with a mass deviation threshold of 5 ppm, signal strength deviation of 30%, and a minimum signal-to-noise ratio of 3. Peak extraction and quantification of target ions were performed based on signal intensity and other relevant criteria. The molecular formulas of detected metabolites were predicted from the molecular ion peaks and fragment ions, then compared with mzCloud, mzVault, and Masslist databases. The original quantitative results were normalized, and the identities and relative levels of metabolites in the serum samples were determined.

### Targeted metabolomics analysis

Targeted metabolomics analysis was conducted using a validated LC–MS/MS method. Serum samples (100 µL) were mixed with 400 µL of pre-chilled methanol solution (80%) and vortexed. The mixture was incubated on ice for 10 min to ensure thorough protein precipitation. Afterward, samples were centrifuged at 15,000×*g* for 10 min at 4 °C, and the supernatants were collected. The supernatant was diluted with a specific solvent to match the desired final methanol concentration and subjected to further centrifugation to remove any remaining particulate matter. Chromatographic separation was performed using a high-performance liquid chromatography (HPLC) system coupled to a triple quadrupole mass spectrometer (QqQ MS). For the separation, a C18 column was utilized at a temperature of 40 °C with a flow rate of 0.2 mL/min. The mobile phase for positive ion mode consisted of 0.1% formic acid in water (A) and methanol (B). In negative ion mode, the mobile phases were 5 mM ammonium acetate in water (A) and methanol (B). The mass spectrometer was operated in multiple reaction monitoring (MRM) mode, with specific transitions selected for each targeted metabolite. Parameters such as collision energy and dwell time were optimized for each analyte to achieve the highest sensitivity. The ion source was operated in both positive and negative ion modes, and the following conditions were set: spray voltage at 3.5 kV, gas flow rate at 10 L/min, and the ion transfer tube temperature at 350 °C. The mass spectrometer was calibrated to ensure precise detection of the metabolites of interest. Data analysis was carried out using specialized software, where peak intensities corresponding to the specific metabolites were integrated and quantified. Each targeted metabolite was identified by its unique retention time and MRM transition. The quantification was based on calibration curves generated from known concentrations of standards. Metabolite concentrations were normalized to internal standards to account for any technical variations during the analysis. This targeted approach enables the quantification of specific metabolites of interest with high accuracy and sensitivity.

### Animals

A total of 30 male SPF-grade Wistar rats, aged 8 weeks and weighing (250 ± 10) g, were purchased from SPF (Beijing) Biotechnology Co., Ltd. Upon arrival at the Animal Facility of Nankai Hospital, rats were acclimated for 7 days under standard laboratory conditions (12-h light/dark cycle, temperature 24 ± 2 °C, relative humidity approximately 60%) with ad libitum access to food and water. All experimental procedures were conducted in strict compliance with the guidelines approved by the Animal Ethics Committee of NanKai Hospital (GENINK-20240056).

### Establishment of RE model

After the adaptation period, rats were randomly assigned into three groups (*n* = 10 per group): sham-operated, model (treatment with PBS), and XDD treatment groups. Except for the sham group, rats at 9 weeks of age underwent surgery to establish the RE model via cardioplasty + pyloric ligation + Roux-en-Y esophagojejunostomy, as previously described by Tang et al. [24]. Briefly, rats were fasted for 24 h before surgery and anesthetized by intraperitoneal injection of 1% sodium pentobarbital (40 mg/kg). A longitudinal incision (~ 0.5 cm) was made at the cardia extending into the esophagus and stomach, then sutured transversely using interrupted 6-0 non-invasive sutures (Shanghai Medical Suture Needle Factory). The pyloric vessels were separated, and the pylorus ligated. The jejunum was transected 8–10 cm distal to the pylorus; the distal end was anastomosed end-to-end with the greater curvature of the glandular stomach, and the proximal end anastomosed end-to-side to the jejunal wall approximately 12–15 cm from the transection site. The abdominal incision was closed in layers post-procedure. To prevent postoperative infection, all operated rats received intramuscular injections of cefoperazone (0.1 g per rat, 0.1 mL/100 g body weight) once daily for 3 consecutive days. A 7-day recovery period was then set to ensure stabilization of the rats’ physiological status and to minimize confounding effects of surgery and antibiotics on subsequent interventions and sample analyses.

Rats in the XDD group received intragastric administration of XDD at a dose of 10 mL/kg once daily for 14 days. Fresh serum samples were collected at the end of treatment, immediately frozen in liquid nitrogen, and stored at − 80 °C for subsequent analysis.

### Preparation of XDD-containing serum

Healthy male SPF-grade Wistar rats (8 weeks old, weighing 250 ± 10 g) were acclimatized for 7 days and then randomly divided into four groups (*n* = 7 per group): low-dose group (XDD-L), medium-dose group (XDD-M), high-dose group (XDD-H), and blank control group. Based on body surface area conversion between humans and rats, the medium dose (XDD-M, 8.55 g/kg) was set as the clinically equivalent dose for rats. The low (XDD-L, 4.275 g/kg) and high (XDD-H, 17.1 g/kg) doses were then derived at ratios of 0.5:1:2 relative to the medium dose. The blank control group received an equal volume of normal saline. All groups were administered twice daily for 3 consecutive days at a gavage volume of 1 mL/100 g body weight. One hour after the final administration, rats were anesthetized by intraperitoneal injection of 10% chloral hydrate, and blood was collected via abdominal aortic puncture. The blood samples were clotted at room temperature for 30 min, followed by centrifugation at 3,000 rpm for 15 min at 4 °C to obtain supernatant serum. The serum was inactivated by water bath at 56 °C for 30 min, sterilized by filtration through a 0.22 μm microporous membrane, aliquoted, and stored at − 20 °C until use to avoid repeated freeze–thaw cycles.

For cell treatment, inflammatory HEECs were incubated with serum-free medium containing 10% (v/v) XDD-containing serum (XDD-L, XDD-M, or XDD-H) or 10% (v/v) blank control serum.

### The differentiation of Th17 cells

Peripheral blood mononuclear cells (PBMCs) were isolated using Ficoll density centrifugation, as described in previous studies [25]. The viability of mononuclear cells, assessed by Trypan Blue exclusion, was consistently greater than 98%. CD4^+^ T lymphocytes were isolated from PBMCs using negative selection, following the manufacturer's instructions (Miltenyi Biotech, San Diego, CA). The purity of the CD4^+^ T cell population was consistently above 92%. After isolation, the cells were resuspended in RPMI 1640 medium, supplemented with 2 mM l-glutamine, 100 IU/mL penicillin, 100 µg/mL streptomycin, 1% non-essential amino acids, and 10% fetal bovine serum (FBS), and cultured under Th17 polarization conditions for five days. According to the previously report [25, 26], these conditions included IL-6 (50 ng/mL), TGF-β (5 ng/mL), IL-1β (25 ng/mL), and Dynabeads Human T Activator (Thermo Fisher Scientific) at a bead-to-cell ratio of 1:50.

### Flow cytometry

The phenotype of in vitro-polarized Th17 cells was characterized by intracellular flow cytometry. Briefly, approximately 2 × 10⁶ cells per sample were first fixed with 4% paraformaldehyde (20 min, room temperature) and then permeabilized with 0.2% Triton X-100 (15 min, 4 °C). After Fc receptor blocking with TruStain FcX, the cells were stained with APC-anti-human CD4 (clone A161A1) and PE-anti-human IL-17A (clone BL168; both from BioLegend). A Zombie Aqua™ viability dye was used to discriminate live/dead cells. Data were acquired on a CytoFLEX LX flow cytometer (Beckman Coulter) and analyzed using FlowJo software (v10.0, Tree Star). The Th17 cell population was defined by gating on single, live CD4⁺ lymphocytes and subsequently assessing the percentage of IL-17A⁺ cells.

### Cell culture

Human normal esophageal epithelial cells (HEEC), were cultured in complete DMEM medium supplemented with 20% fetal bovine serum (FBS) under conditions of 37 °C and 5% CO₂, with humidity maintained at saturation to ensure optimal growth and proliferation of the cells.

### Induction of inflammatory phenotype

To induce an inflammatory phenotype in HEEC cells, a stimulatory microenvironment was established. The culture medium was acidified to pH 5.0 to mimic an acidic milieu. Concurrently, the cells were treated with 100 μM deoxycholic acid (DCA) to potentiate the inflammatory response. This combined treatment of low pH and DCA was designed to simulate pathological changes characteristic of esophageal epithelial tissue.

### l-Kynurenine treatment

In vitro polarized Th17 cells were exposed to 200 μM l-kynurenine for the final 36 h of culture. The concentration and duration of treatment were determined based on our previously established and validated protocols [25, 26]. As per the manufacturer's instructions and prior reports [7], l-kynurenine was reconstituted in 0.5 M HCL prior to application.

### AHRR gene silencing

In additional experiments, both untreated and l-kynurenine-treated Th17 cells were subjected to AHRR silencing using Silencer Select siRNA (Thermo Fisher Scientific). Cells were resuspended in Opti-MEM medium (Thermo Fisher Scientific) and seeded at a density of 2.5 × 10^5^ cells per well in 96-well plates. AHRR-specific siRNA was added at a final concentration of 1 pmol per well during the last 18 h of culture. A random RNA (Thermo Fisher Scientific) was used as a negative control. AHRR silencing was confirmed via quantitative PCR (qPCR) using specific gene primers, with details provided below. In separate experiments, CD4^+^ T cells isolated by immunomagnetic separation (Miltenyi Biotech) were exposed to AHRR siRNA or random RNA on day 1 and day 3 of Th17 polarization. Subsequently, expression levels of IL-17A and RORC genes were measured.

### AHRR overexpression

CD4⁺ T cells were differentiated into Th17 cells and then transfected with AHRR-OE or empty vector using Lipofectamine™ 3000 reagent following the manufacturer’s protocol. Six hours later, cells were treated with HEEC-sup or KYN for 24 h and harvested for RT-qPCR and flow cytometry analysis.

### CCK-8 assay

Cell viability was assessed using a Cell Counting Kit-8 (CCK-8). HEEC cells were seeded in 96-well plates at a density of 1 × 10^4^ cells per well in 100 μL of complete medium and allowed to adhere for 24 h. The cells were then treated with acidic medium (pH 5.0) containing deoxycholic acid (DCA) at various concentrations (50, 100, 200, 300 μM) for different durations (2, 4, 6, 8 h). Following treatment, the medium was replaced with 100 μL of CCK-8 working solution, and the plates were incubated at 37 °C for 2 h. The absorbance at 450 nm was finally measured using a microplate reader. All conditions were assayed in triplicate wells.

### H&E staining

Rat esophageal tissues were fixed in 4% paraformaldehyde, dehydrated with graded ethanol, embedded in paraffin, and sectioned. The paraffin sections were stained with H&E and examined under a microscope (Olympus, Tokyo, Japan).

### Real-time quantitative PCR

Total RNA was extracted from cells and colon tissues using TRIzol reagent (Invitrogen) according to the manufacturer's instructions. Complementary DNA (cDNA) was synthesized from the extracted RNA using the Hifair^®^ III First Strand cDNA Synthesis Kit (Yeasen Biotech). Quantitative real-time PCR (qRT-PCR) was performed using the Hieff^®^ qPCR SYBR Green system and the LightCycler 480 SYBR Green I Master Mix (Roche) following the manufacturer’s recommendations. Gene expression levels were normalized to GAPDH and quantified using the 2^−ΔΔC*t*^ method. The sequences of the primers used are listed in Additional file 1: Table S1.

### ELISA

Following the manufacturer's protocol, samples and standards were prepared and introduced into the wells of a microtiter plate, which had been pre-coated with a primary antibody specific to the target protein. The plate was incubated at 37 °C for 1 h, followed by a washing step to eliminate any unbound substances. After washing, 50 µL of substrate A and substrate B were added to each well, and the plate was incubated in the dark at 37 °C for 15 min. The reaction was then terminated using a stop solution, and optical density at 450 nm was measured using a microplate reader.

#### Statistical analysis

Data derived from all experimental procedures were analyzed using GraphPad Prism 9.0 software. The Shapiro–Wilk test was employed to assess data normality, while the Levene's test evaluated homogeneity of variance. The data were analyzed by two-tailed unpaired Student’s *t*-test or one-way ANOVA analysis. Non-normally distributed or heteroscedastic data were subjected to non-parametric Mann–Whitney *U* tests. For multiple group comparisons, Tukey's post-hoc analysis was conducted following one-way ANOVA analysis. All the data are presented as the means ± standard errors of the means (SEMs) from at least three independent duplications. *P* < 0.05 was considered statistically significant.

## Results

### Elevated IL-17A levels correlate with RE disease severity

**Fig. 1:**
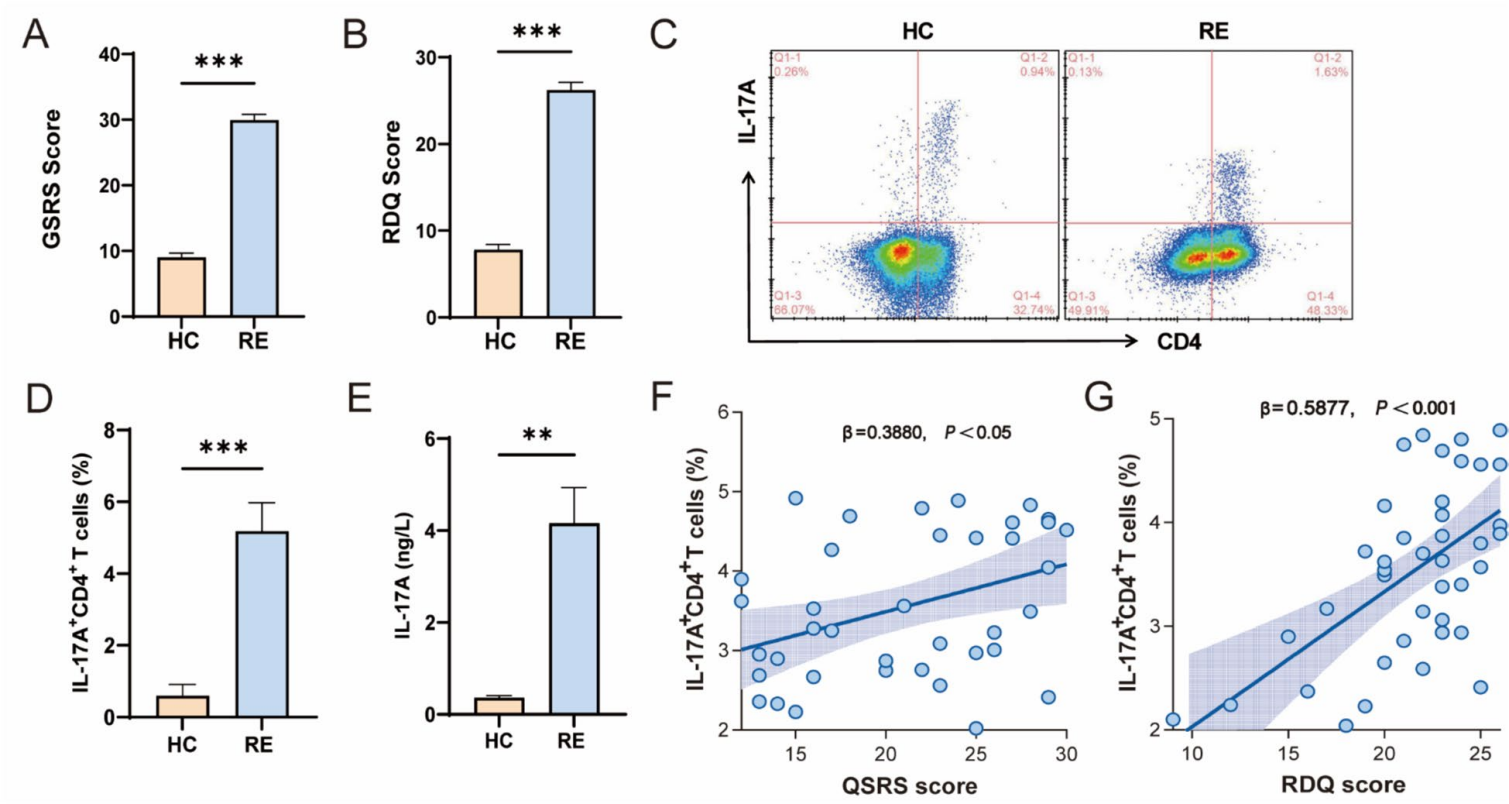
Elevated IL-17A levels correlate with RE disease severity. **A** Gastroesophageal Reflux Disease Symptom Scale (GSRS) score in healthy controls (*n* = 40) and patients with reflux esophagitis (RE) (*n* = 40). **B** Reflux Disease Questionnaire (RDQ) score in healthy controls and RE patients. **C, D** Proportion of IL-17A⁺ CD4⁺ T cells in peripheral blood of healthy controls (*n* = 20) and RE patients (*n* = 20) measured by flow cytometry. **E** IL-17A levels in peripheral blood from healthy controls and RE patients measured by ELISA. **F** Correlation between IL-17A expression and GSRS score. **G** Correlation between IL-17A expression and RDQ score. Statistical significance was determined using independent Student's *t* test after assessing variance homogeneity with Levene’s test (**A, B, D, E**). All data are presented as the mean ± SEMs. *P < 0.05, **P < 0.01, ***P < 0.001

To determine the association between Th17-mediated immunity and RE, we first compared clinical scores and IL-17A levels between RE patients and healthy controls. Patients with RE exhibited significantly higher Gastroesophageal Reflux Disease Symptom Scale (GSRS) and Reflux Disease Questionnaire (RDQ) scores than healthy individuals (Fig. 1A, B), confirming greater symptom severity. At the cellular level, flow cytometry analysis revealed a marked increase in the frequency of IL-17A-producing cells in RE patients (Fig. 1C, D). Consistent with this, ELISA results demonstrated that IL-17A levels in the serum were also significantly elevated in the RE group compared to controls (Fig. 1E). Importantly, correlation analysis indicated a positive relationship between serum IL-17A levels and both GSRS (Fig. 1F) and RDQ scores (Fig. 1G). These results demonstrated that IL-17A levels are heightened in RE and are positively correlated with disease severity.

### Metabolomic profiling identifies tryptophan pathway dysregulation in RE​

**Fig. 2:**
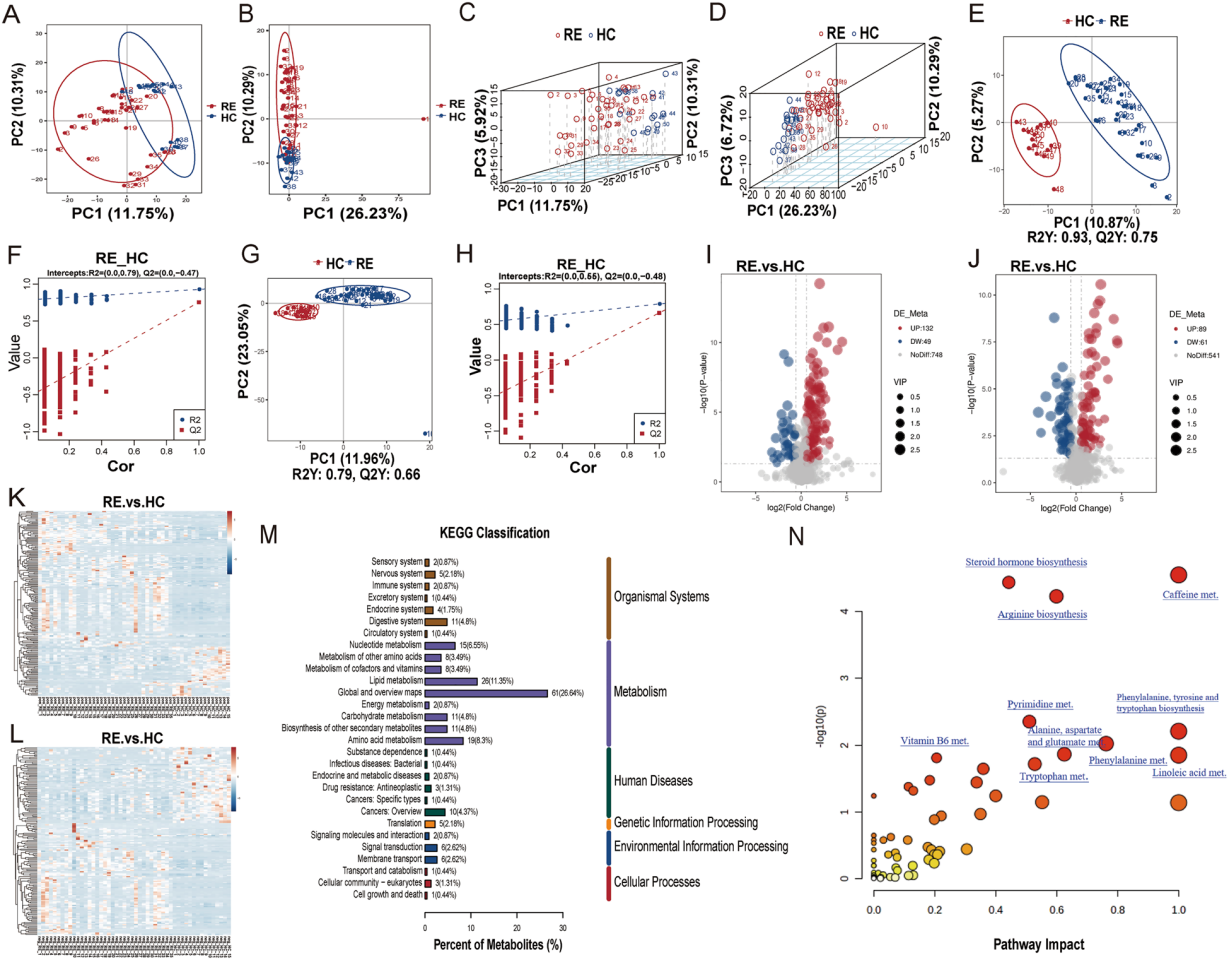
Metabolomic profiling identifies tryptophan pathway dysregulation in RE. **A**–**D** Untargeted metabolomics was performed to compare the serum metabolic profiles of between healthy controls (*n* = 15) and patients with RE (*n* = 35). Principal component analysis (PCA) score plots of metabolites detected in positive ion mode (**A, C**) and negative ion mode (**B, D**). **E**–**H** Partial least squares-discriminant analysis (PLS-DA) score plots in positive ion mode (**E, F**) and negative ion mode (**G, H**). **I, J** Volcano plots of differential metabolites under positive (**I**) and negative (**J**) ion mode. **K, L** Hierarchical clustering heatmaps of differential metabolites in positive (**K**) and negative (**L**) ion modes. **M **KEGG pathway enrichment analysis of differentially abundant metabolites. **N** Topological analysis of altered metabolic pathways

To systemically characterize the metabolic perturbations in RE, we performed untargeted metabolomic profiling of serum samples from RE patients and healthy controls using ultra-high-performance liquid chromatography-coupled with high-resolution mass spectrometry (UHPLC-MS). After excluding isotope peaks, a total of 929 metabolites were identified in positive ion mode, and 691 metabolites were identified in negative ion mode. Unsupervised principal component analysis (PCA) was conducted to assess the intrinsic metabolic variations and data quality. The results revealed a clear separation of metabolites between two groups, indicating distinct global metabolic profiles (Fig. 2A–D).

We further employed a supervised partial least squares-discriminant analysis (PLS-DA), which yielded robust models without overfitting, and confirmed pronounced metabolic discrimination between RE patients and controls in both positive and negative ion modes (Fig. 2E–H). Comparative analysis identified numerous significantly dysregulated metabolites: 184 in positive ion mode (135 upregulated, 49 downregulated) and 156 in negative ion mode (92 upregulated, 64 downregulated) (Fig. 2I, J). Unsupervised hierarchical clustering of these differential metabolites revealed distinct patient-specific patterns (Fig. 2K, L).

KEGG pathway enrichment analysis of the altered metabolites highlighted significant involvement in lipid, amino acid, and nucleotide metabolism (Fig. 2M). Crucially, topological analysis identified the tryptophan metabolism and biosynthesis pathway as one of the most significantly enriched (Fig. 2N). These data underscore systemic metabolic reprogramming in RE and specifically implicate aberrant tryptophan metabolism as a key component of the disease's pathophysiology.

### Targeted metabolomics confirms dysregulation of the kynurenine pathway in RE

**Fig. 3:**
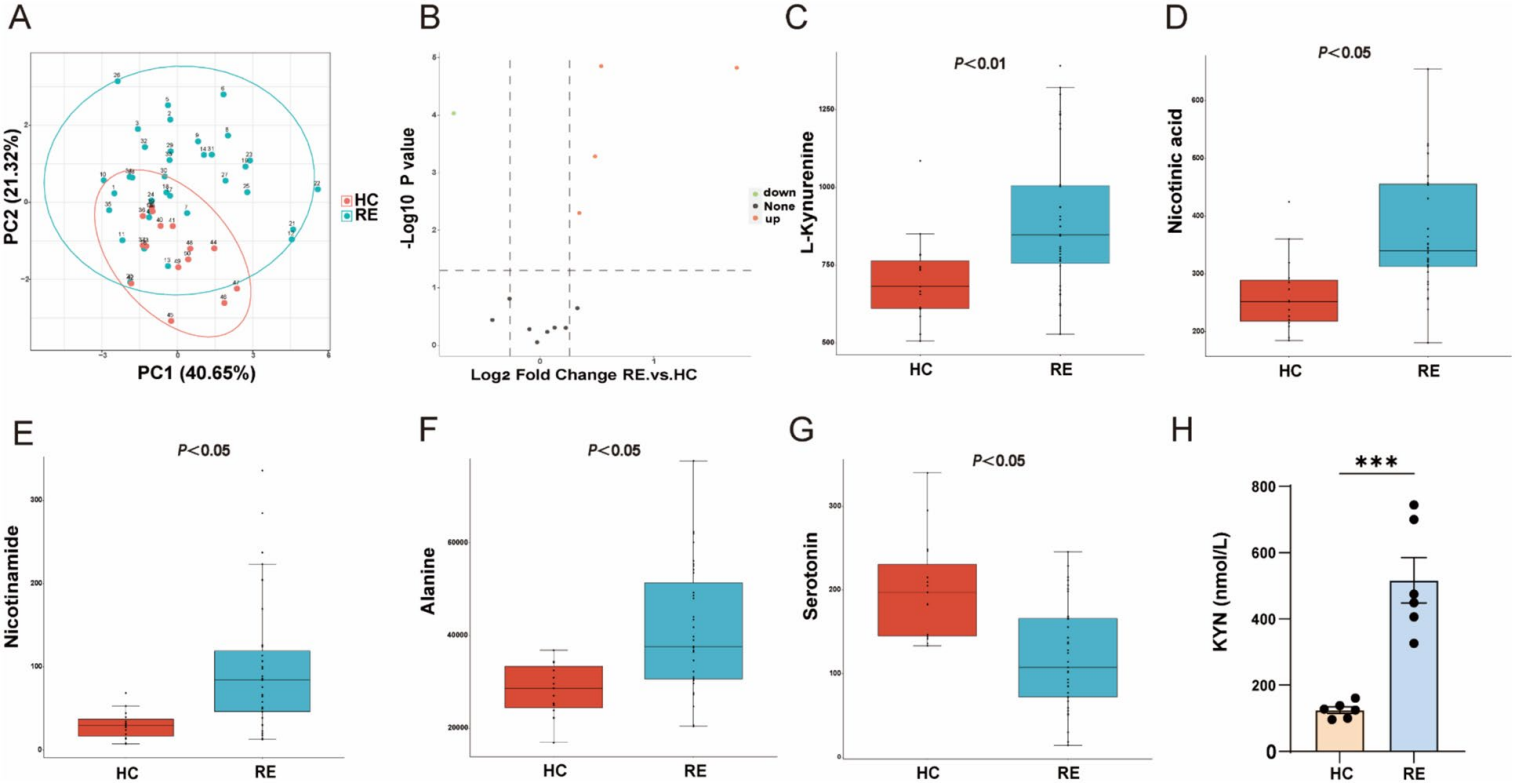
Targeted metabolomics confirms dysregulation of the kynurenine pathway in RE. **A** Principal component analysis (PCA) of serum metabolites from patients with RE (*n* = 35) and healthy controls (HC) (*n* = 15). **B** Volcano plot of differentially abundant metabolites in the serum of RE patients compared to HC. **C**–**G** Altered levels of tryptophan metabolites in RE patients: elevated levels of kynurenine (**C**), niacin (**D**), nicotinamide (**E**), and alanine (**F**), and reduced level of serotonin (**G**). **H** Detection of KYN concentration in serum from healthy controls and RE patients measured by ELISA (*n* = 6). All data are presented as the mean ± SEMs. Statistical significance was determined using independent Student's *t* test after assessing variance homogeneity with Levene’s test. *P < 0.05, **P < 0.01, ***P < 0.001

To validate the differences in tryptophan metabolism between patients with RE and healthy controls, we conducted a targeted metabolomic analysis. The results revealed a significant difference in the metabolic profiles between the RE patients and the healthy control group, as evidenced by clear separation in PCA (Fig. 3A) and the dysregulation of five key metabolites (Fig. 3B).

Notably, the metabolites consistently elevated in RE patients—kynurenine, niacin, nicotinamide, and alanine (Fig. 3C–F)—are integral components of the kynurenine pathway. In contrast, serotonin, a metabolite of the competing alternative pathway, was significantly reduced in RE patients (Fig. 3G). The results of ELISA analysis showed a significant increase in the level of kynurenine in the serum of RE patients compared to controls (Fig. 3H).

Collectively, these results define a distinct shift in tryptophan metabolism in RE, characterized by a prominent activation of the kynurenine pathway. This observation further suggests that the tryptophan metabolic pathway may serve as a critical mediator in RE pathogenesis.

### Kynurenine pathway activation and esophageal inflammation are confirmed in a rat model

**Fig. 4:**
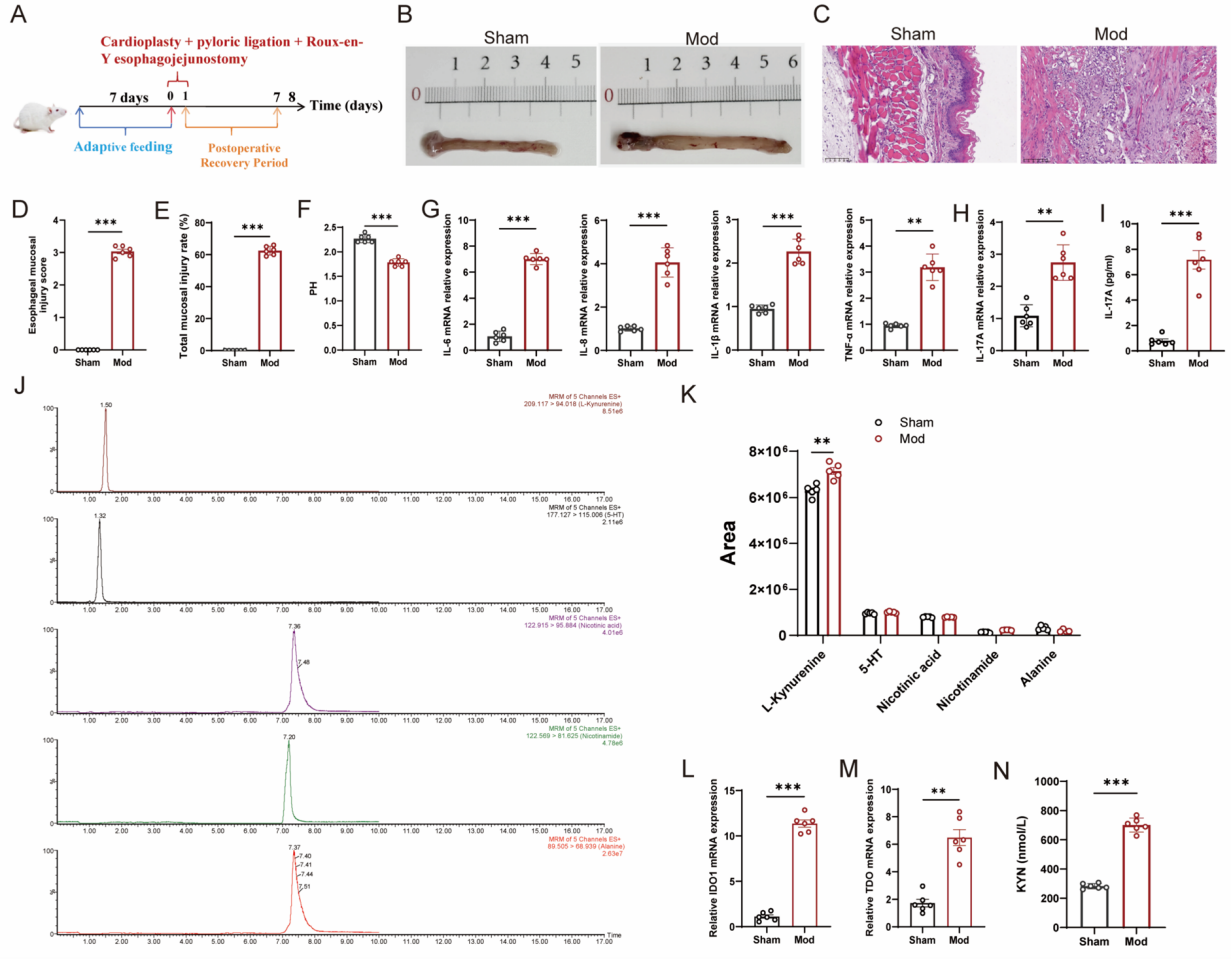
Kynurenine pathway activation and esophageal inflammation are confirmed in a rat model. **A** Schematic of the experimental design for establishing the rat RE model (*n* = 6 per group). **B** The extent of esophageal injury in each group. **C** Representative images of hematoxylin and eosin (HE) staining. **D** Esophageal mucosal injury score. **E** Total mucosal injury rate. **F** The esophageal PH levels in different groups. **G, H** mRNA expression of IL-6, IL-8, IL-1β, TNF-α, and IL-17A in esophageal tissues. **I** IL‑17A levels in serum from healthy controls and RE rats measured by ELISA. **J, K** Detection of l-kynurenine, niacin, nicotinamide, alanine, and serotonin levels in rat serum using liquid chromatography. **L, M** mRNA levels of IDO1 (**L**) and TDO (**M**) in rat esophageal tissues (*n* = 6 per group). **N** Detection of serum KYN levels in sham and RE model rats by ELISA. All data are presented as the mean ± SEMs. Statistical significance was determined using independent Student's t test after assessing variance homogeneity with Levene’s test. *P < 0.05, **P < 0.01, ***P < 0.001

To validate our clinical findings in a controlled experimental system, we employed a rat model of RE (Fig. 4A). Successful induction of esophagitis was confirmed by histopathological assessment, which revealed significant mucosal injury, including erosions, congestion, edema and inflammatory cell infiltration in RE model rats, compared to the normal esophageal architecture observed in Sham-operated controls (Fig. 4B, C). The injury severity score and total mucosal injury rate were significantly higher in the model group (Fig. 4D, E). A decrease in esophageal pH confirmed an acidic microenvironment in RE rats (Fig. 4F), mimicking a key clinical feature.

Molecular analysis of esophageal tissue demonstrated a robust inflammatory response in RE rats, characterized by significantly upregulated mRNA expression of pro-inflammatory cytokines (IL-6, IL-8, IL-1β, TNF-α) (Fig. 4G), notably, IL-17A (Fig. 4H). Elevated IL-17A protein levels were further confirmed by ELISA (Fig. 4I), reinforcing the relevance of the Th17 axis in this model.

Critically, liquid chromatography analysis of five kynurenine metabolites in serum revealed a significant upregulation of kynurenine in RE rats group (Fig. 4J, K), indicating kynurenine metabolic abnormalities are closely associated with the development of RE. This metabolic shift was associated with a marked upregulation at the enzymatic level, as evidenced by increased mRNA expression of the key tryptophan-catabolizing enzymes IDO1 and TDO in the model group (Fig. 4L, M). The elevated kynurenine levels were further validated by ELISA (Fig. 4N), suggesting the involvement of kynurenine metabolism in the pathogenesis of RE.

Collectively, these data demonstrate that the RE rat model faithfully recapitulates the core pathophysiological features of the human disease, including kynurenine pathway activation, and positions kynurenine as a key metabolite linking tryptophan metabolism to esophageal inflammation.

### HEEC-derived kynurenine promotes Th17 cell differentiation

**Fig. 5:**
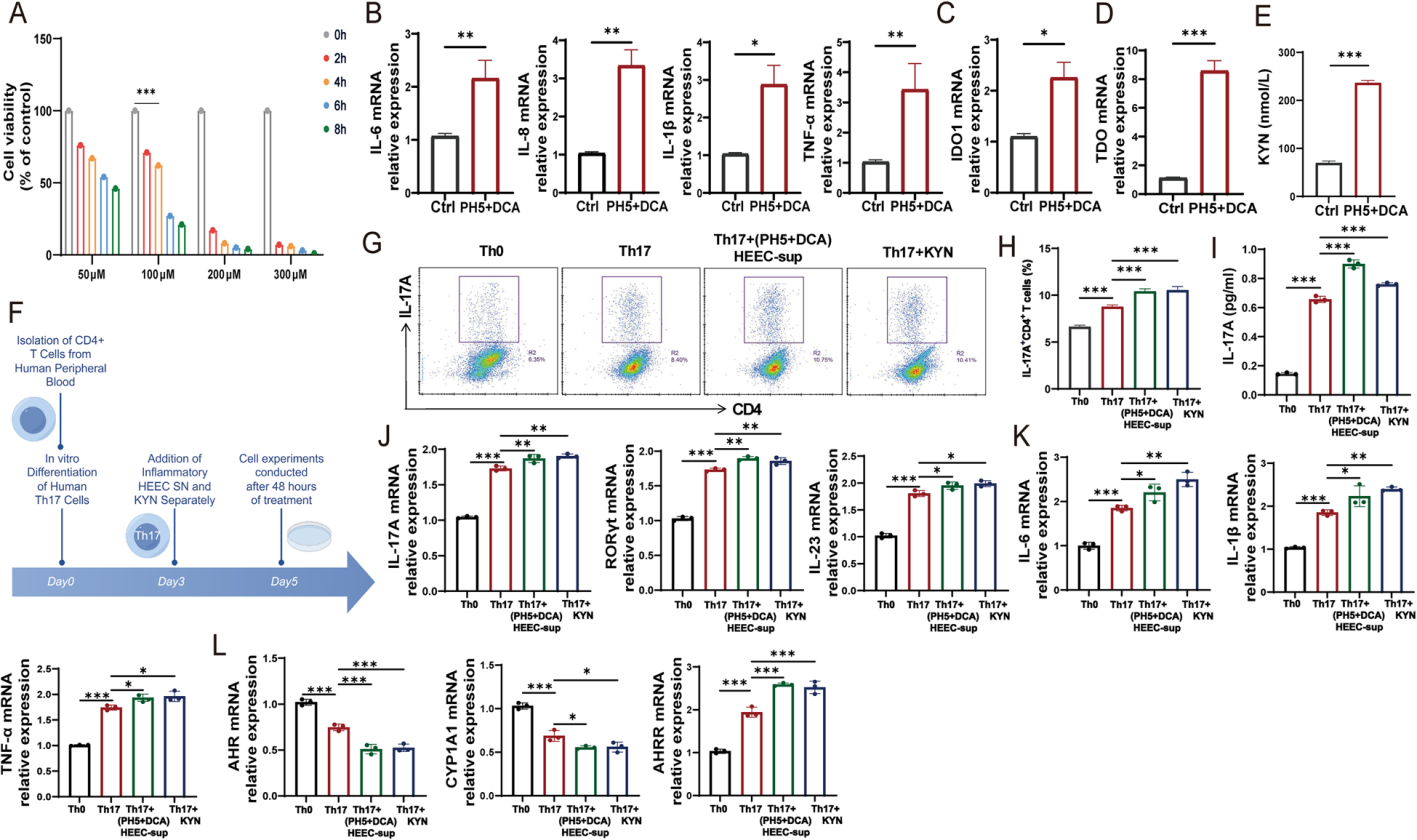
HEEC-derived kynurenine promotes Th17 cell differentiation. **A** Viability of HEECs following stimulation with acidic DCA solutions (50, 100, 200, and 300 μM) for 2, 4, 6, and 8 h, measured using the CCK-8 assay (*n* = 3; 0 h as control). **B–D** mRNA levels of inflammatory cytokines (IL-6, IL-8, IL-1β, TNF-α) **B**, IDO1 **C**, and TDO **D** in HEEC. **E** KYN concentration in cell supernatant measured by ELISA. **F **Schematic diagram of in vitro Th17 differentiation from CD4⁺ T cells. CD4^+^ T cells isolated from healthy peripheral blood mononuclear cells (PBMCs, *n* = 3) were differentiated under standard Th0 or Th17 cell conditions, with or without conditioned medium from (pH 5.0 + DCA)-stimulated HEECs or exogenous KYN (*n* = 3). **G, H** Flow cytometry analysis of IL‑17A expression in CD4⁺ T cell. **I** IL‑17A levels in cell supernatant measured by ELISA. **J–L** mRNA expression of cytokines (IL-17A, RORγt, IL-23, IL-6, IL-1β, TNF-α) and AhR pathway genes (AHR, CYP1A1, AHRR) in the Th17 cell. All data are presented as the mean ± SEMs of three independent experiments (*n* = 3 per group). Statistical significance was determined using independent Student's *t* test after assessing variance homogeneity with Levene’s test. *P < 0.05, **P < 0.01, ***P < 0.001

Given that esophageal epithelial cells represent the primary site of injury in RE, we hypothesized that these cells serve as a major source of KYN, which in turn directly drives Th17-cell-mediated inflammation. To test this, we first established an inflammatory model using HEECs by screening various stimuli. A 4-h treatment with the combination of pH 5.0 and 100 µM DCA was identified as the optimal condition and was used to induce a robust inflammatory response, as evidenced by decreased cell viability (Fig. 5A) and significant upregulation of IL-6, IL-8, IL-1β, and TNF-α mRNA expression (Fig. 5B).

Under these inflammatory conditions, HEECs exhibited increased expression of the kynurenine pathway enzymes IDO1 and TDO (Fig. 5C, D), leading to elevated KYN production (Fig. 5E). To assess the functional impact of HEEC-derived KYN on Th17 cells, we cultured pre-differentiated Th17 cells with conditioned medium from inflamed HEECs (HEEC-sup) or exogenous KYN (Fig. 5F). Flow cytometry and ELISA analyses revealed that both HEEC-sup and exogenous KYN significantly enhanced IL-17A production in Th17 cells compared to the Th17 control group (Fig. 5G–I). This pro-inflammatory effect was associated with upregulated expression of key Th17-related genes (IL-17A, RORγt, IL-23, IL-6, IL-1β, TNF-α) and AHRR, alongside downregulation of AHR and its target gene CYP1A1 (Fig. 5J–L). These results demonstrate that KYN promotes Th17 response.

**Fig. 6:**
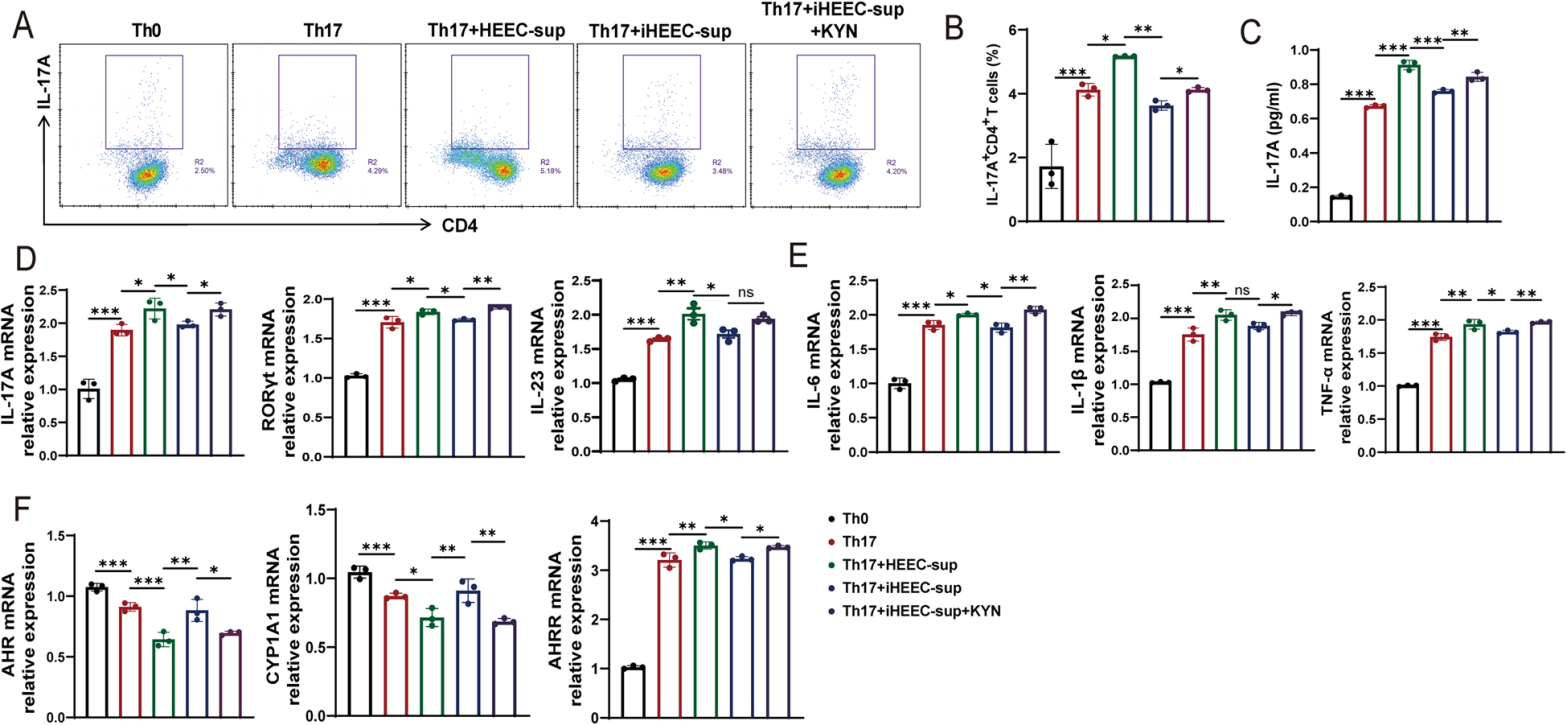
Kynurenine derived from HEEC promotes Th17 cell activation. **A, B** CD4^+^ T cells isolated from healthy donor PBMCs were differentiated into Th17 cells, and then treated with supernatant from inflammatory HEEC cells (HEEC-sup) or HEECs pretreated with an IDO1/TDO inhibitor (iHEEC-sup), or iHEEC-sup supplemented with exogenous KYN (iHEEC-sup + KYN). Intracellular IL-17A expression in CD4^+^ T cells was assessed by flow cytometry on day 5. **C** IL-17A concentration in cell culture supernatant was measured by ELISA. **D–F** mRNA expression of Th17-related cytokines (IL-17A, RORγt, IL-23, IL-6, IL-1β, TNF-α) and AhR pathway genes (AHR, CYP1A1, AHRR) in Th17 cells (*n* = 3). All data are presented as the mean ± SEMs of three independent experiments. Statistical significance was determined using one-way ANOVA with Tukey's post hoc test. *P < 0.05, **P < 0.01, ***P < 0.001

To determine the role of HEEC-derived KYN in Th17 activity, we performed a rescue experiment. Th17 cells were cultured with conditioned medium from (IDO + TDO) inhibitor treated-inflamed HEECs. Flow cytometry and ELISA analysis showed that inhibition of IDO1/TDO in HEECs abrogated the ability of HEEC-sup to enhance IL-17A production in Th17 cells, Critically, this effect was reversed by the addition of exogenous KYN (Fig. 6A–C). Concomitant changes in the expression of Th17-related genes (IL-17A, RORγt, IL-23, IL-6, IL-1β, TNF-α) and AhR pathway gene (AHR, AHRR, CYP1A1) were consistently observed (Fig. 6D–F). These data demonstrate that KYN derived from inflamed esophageal epithelium directly promotes Th17 cell activation.

### AHRR silencing abrogates the kynurenine-mediated enhancement of Th17 differentiation

**Fig. 7:**
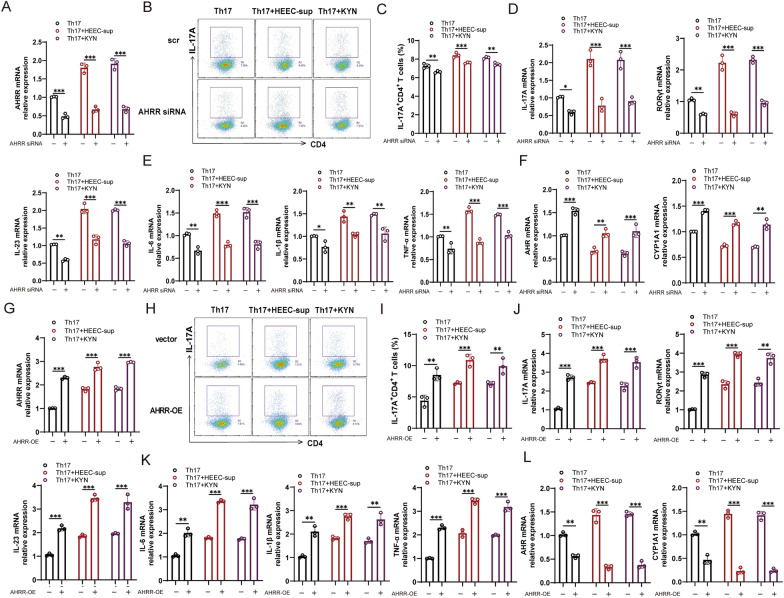
AHRR Silencing Abrogates the Kynurenine -Mediated Enhancement of Th17 activation. **A–C** CD4^+^ T cells isolated from healthy donor PBMCs were differentiated into Th17 cells, and then transfected with AHRR siRNA or scramble control (scr) siRNA for 24 h. Cells were subsequently treated with supernatant from inflammatory HEEC cell (HEEC-sup) or exogenous KYN. **A** mRNA levels of AHRR in the Th17 cell (*n* = 3). **B, C** Flow cytometry and statistical analysis of IL-17A^+^ cells. **D–F** mRNA expression of Th17-related genes (IL-17A, RORγt, IL-23, IL-6, IL-1β, TNF-α) and AhR pathway genes (AHR, CYP1A1) in the Th17 cells (*n* = 3). **G–I** CD4⁺ T cells isolated from healthy donor PBMCs were differentiated into Th17 cells, and then transfected with AHRR overexpression plasmid (AHRR-OE) or empty vector control (vector) for 24 h. Cells were subsequently treated with supernatant from inflammatory HEEC cell (HEEC-sup) or exogenous KYN. **A** mRNA levels of AHRR in the Th17 cells (*n* = 3). **H, I** Flow cytometry and statistical analysis of IL-17A⁺ cells. **J–L** mRNA expression of Th17-related genes (IL-17A, RORγt, IL-23, IL-6, IL-1β, TNF-α) and AhR pathway genes (AHR, CYP1A1) in the Th17 cells (*n* = 3). All data are presented as the mean ± SEMs of three independent experiments. Statistical significance was determined using two-way ANOVA with Tukey's post hoc test. *P < 0.05, **P < 0.01, ***P < 0.001

Given the observed inverse relationship between AHRR and AHR signaling in KYN-exposed Th17 cells, we hypothesized that AHRR upregulation is a critical mechanism by which kynurenine exacerbates Th17-driven inflammation. To test this, we first silenced AHRR expression in Th17 cells using siRNA (Fig. 7A) and assessed their functional response.

Flow cytometry analysis revealed that AHRR knockdown significantly attenuated the enhanced IL-17A production induced by both HEEC-conditioned medium (HEEC-sup) and exogenous KYN (Fig. 7B, C). This reversal of the pro-inflammatory phenotype was further corroborated at the transcriptional level. Silencing AHRR led to a marked downregulation of key Th17-related genes, including IL-17A, the transcription factor RORγt, and the pro-inflammatory cytokines IL-23, IL-6, IL-1β, and TNF-α (Fig. 7D, E). In contrast, the expression of AHR and CYP1A1 was upregulated (Fig. 7F).

To further validate the regulatory role of AHRR, we overexpressed AHRR in Th17 cells via transfection with an AHRR overexpression plasmid (AHRR-OE) (Fig. 7G). Compared with the empty vector control, AHRR overexpression markedly amplified the promotive effects of HEEC-sup and exogenous KYN on IL-17A expression (Fig. 7H, I), while simultaneously upregulating the mRNA levels of RORγt, IL-17A, IL-23, IL-6, IL-1β, and TNF-α (Fig. 7J, K). Conversely, overexpression of AHRR further suppressed the expression of AHR and CYP1A1 (Fig. 7L), strengthening the inhibition of AhR signaling.

Collectively, these loss-of-function and gain-of-function data demonstrate that AHRR is essential for the kynurenine-mediated activation of Th17 cells. Kynurenine promotes Th17 inflammation primarily through an AHRR-dependent suppression of AhR signaling.

### XDD alleviates RE by activating AhR and inhibiting Th17 response

**Fig. 8:**
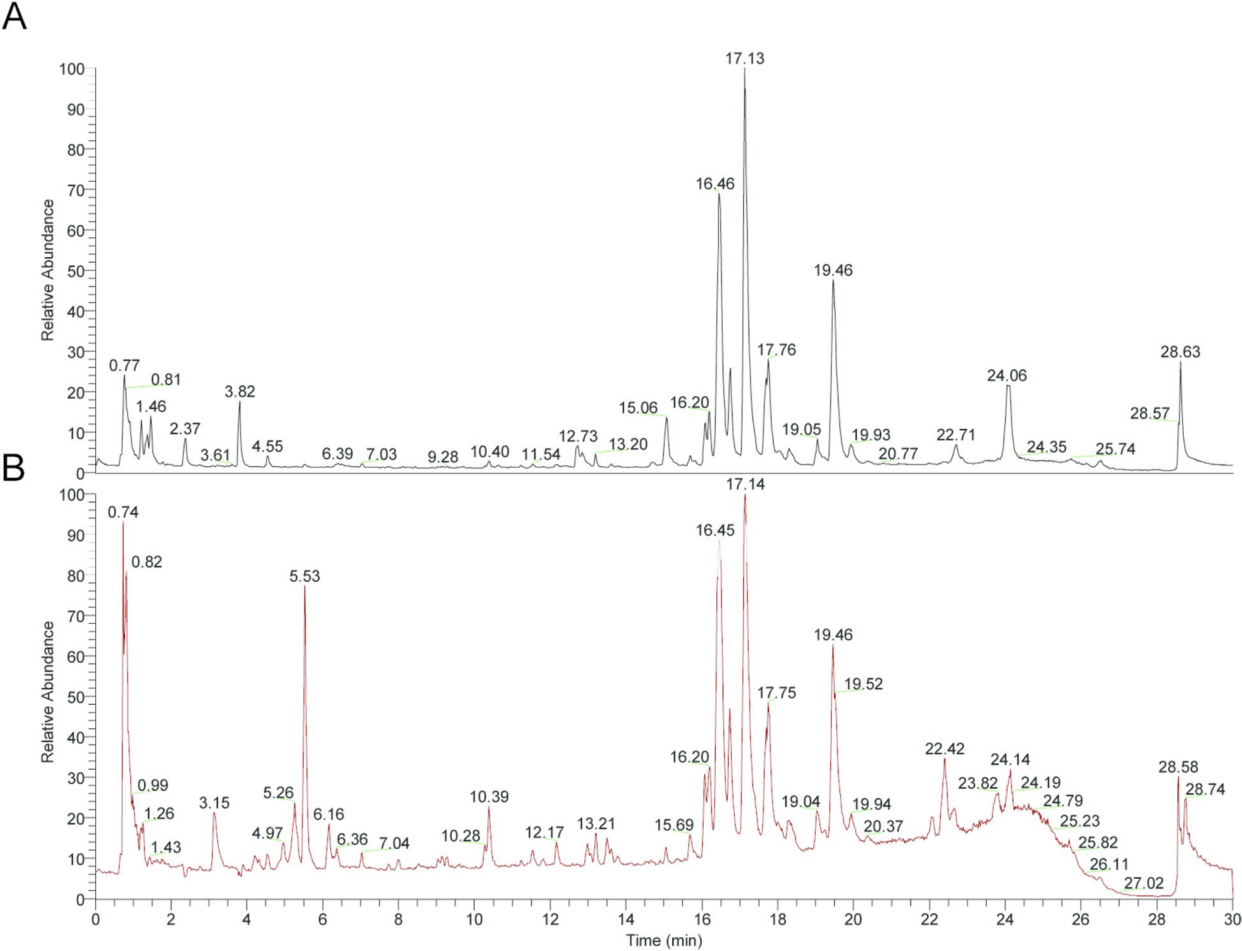
The HPLC fingerprints of XDD as well as its major components. **A** Positive mode. **B** Negative mode

Representative HPLC results are illustrated in (Fig. 8; Tables 2, 3). The main components in the XDD are 6-Gingerol, Neoliquiritin, Glycyrrhizic acid, Liquiritin, Licorice saponin G2, Melliferone, (18β,20α)-Glycyrrhizic acid, Isomer of Melliferone.

**Table 2:** Chemical composition analysis of XDD through HPLC

No	Identification	t_*R*_ (min)	Formula	Observed mass (Da)	Calculated mass (Da)	Mass error (ppm)	Adducts
1	DL-Arginine	0.72	C_6_H_14_N_4_O_2_	175.1197	175.1190	4.00	[M + H]^+^
2	D(+)-Glucose	0.78	C_6_H_12_O_6_	179.0562	179.0561	0.56	[M − H]^−^
3	D-(−)-Mannitol	0.79	C_6_H_14_O_6_	181.0720	181.0718	1.10	[M − H]^−^
4	Valine	0.81	C_5_H_11_NO_2_	118.0866	118.0863	2.54	[M + H]^+^
5	α,α-Trehalose	0.83	C_12_H_22_O_11_	341.1106	341.1089	4.98	[M − H]^−^
6	Proline	0.84	C_5_H_9_NO_2_	116.0710	116.0706	3.45	[M + H]^+^
7	Sucrose	0.88	C_12_H_22_O_11_	341.1106	341.1089	4.98	[M − H]^−^
8	L-(−)-Malic acid	0.91	C_4_H_6_O_5_	133.0148	133.0142	4.51	[M − H]^−^
9	Betaine	0.97	C_5_H_11_NO_2_	118.0867	118.0863	3.39	[M + H]^+^
10	Adenine	1.17	C_5_H_5_N_5_	136.0623	136.0618	3.67	[M + H]^+^
11	Citric acid	1.21	C_6_H_8_O_7_	191.0206	191.0197	4.71	[M − H]^−^
12	Adenosine	1.22	C_10_H_13_N_5_O_4_	268.1053	268.104	4.85	[M + H]^+^
13	Uridine	1.24	C_9_H_12_N_2_O_6_	243.0634	243.0623	4.53	[M − H]^−^
14	L-Tyrosine	1.32	C_9_H_11_NO_3_	182.0819	182.0812	3.84	[M + H]^+^
15	Guanosine	1.34	C_10_H_13_N_5_O_5_	284.1003	284.0989	4.93	[M + H]^+^
16	Fumaric acid	1.41	C_4_H_4_O_4_	115.0041	115.0037	3.48	[M − H]^−^
17	Succinic acid	1.42	C_4_H_6_O_4_	117.0195	117.0193	1.71	[M − H]^−^
18	Isoleucine	1.42	C_6_H_13_NO_2_	132.1024	132.1019	3.78	[M + H]^+^
19	Gallic acid	1.89	C_7_H_6_O_5_	169.0148	169.0142	3.55	[M − H]^−^
20	L-Phenylalanine	2.30	C_9_H_11_NO_2_	166.0870	166.0862	4.82	[M + H]^+^
21	Codonopsinol B	2.75	C_13_H_19_NO_4_	254.1398	254.1387	4.33	[M + H]^+^
22	Codonopsine	2.96	C_14_H_21_NO_4_	268.1555	268.1543	4.48	[M + H]^+^
23	Vanillic acid	2.98	C_8_H_8_O_4_	167.0358	167.0350	4.79	[M − H]^−^
24	Protocatechuic acid	3.45	C_7_H_6_O_4_	153.0198	153.0193	3.27	[M − H]^−^
25	Gentisic acid	3.48	C_7_H_6_O_4_	153.0197	153.0193	2.61	[M − H]^−^
26	Neochlorogenic acid	3.70	C_16_H_18_O_9_	353.0888	353.0878	2.83	[M − H]^−^
27	Tryptophan	3.92	C_11_H_12_N_2_O_2_	205.0981	205.0972	4.39	[M + H]^+^
28	Syringin	4.51	C_17_H_24_O_9_	390.1751	390.1759	-2.05	[M + NH_4_]^+^
29	Chlorogenic acid	4.62	C_16_H_18_O_9_	353.0872	353.0878	-1.70	[M − H]^−^
30	Quinic acid	4.64	C_7_H_12_O_6_	191.0565	191.0561	2.09	[M − H]^−^
31	Cryptochlorogenic acid	4.80	C_16_H_18_O_9_	353.0881	353.0878	0.85	[M − H]^−^
32	Phallodendrin	5.20	C_20_H_24_NO_4_^+^	342.1716	342.1711	1.46	[M]^+^
33	Daphnetin	5.20	C_9_H_6_O_4_	179.0347	179.0339	4.47	[M + H]^+^
34	Caffeic acid	5.29	C_9_H_8_O_4_	179.0355	179.0350	2.79	[M − H]^−^
35	Shaftoside	5.85	C_26_H_28_O_14_	565.1568	565.1552	2.83	[M + H]^+^
36	5-Feruoylquinic acid	5.91	C_17_H_20_O_9_	367.1052	367.1035	4.63	[M − H]^−^
37	Quercetagitrin	6.05	C_21_H_20_O_13_	479.0850	479.0831	3.97	[M + H]^+^
38	Isoshaftoside	6.07	C_26_H_28_O_14_	565.1567	565.1552	2.65	[M + H]^+^
39	Violanthin	6.07	C_27_H_30_O_14_	579.1730	579.1708	3.80	[M + H]^+^
40	Magnocurarine	6.16	C_19_H_24_NO_3_^+^	314.1764	314.1751	4.14	[M]^+^
41	Atractylenolide-1	6.45	C_15_H_18_O_2_	231.1389	231.1380	3.89	[M + H]^+^
42	AtractylenolideⅢ	6.45	C_15_H_18_O_2_	249.1497	249.1485	4.82	[M + H]^+^
43	Rutin	6.57	C_27_H_30_O_16_	609.1489	609.1461	4.60	[M − H]^−^
44	(+)-Magnoflorine	6.60	C_20_H_24_NO_4_^+^	342.1714	342.1711	0.88	[M]^+^
45	Isoliquiritin	6.64	C_21_H_22_O_9_	417.1192	417.1191	0.24	[M − H]^−^
46	Isoviolanthin	6.71	C_27_H_30_O_14_	579.1726	579.1708	3.11	[M + H]^+^
47	Umbelliferone	6.75	C_9_H_6_O_3_	163.0396	163.0390	3.68	[M + H]^+^
48	Liquiritin	6.77	C_21_H_22_O_9_	417.1198	417.1191	1.68	[M − H]^−^
49	Hyperin	6.88	C_21_H_20_O_12_	463.0895	463.0882	2.81	[M − H]^−^
50	Quercetin	6.90	C_15_H_10_O_7_	303.0511	303.0499	3.96	[M + H]^+^
51	Isoquercetin	7.06	C_21_H_20_O_12_	463.0901	463.0882	4.10	[M − H]^−^
52	Isorhamnetin	7.62	C_16_H_12_O_7_	317.0670	317.0656	4.42	[M + H]^+^
53	Britannin	7.62	C_19_H_26_O_7_	367.1769	367.1751	4.90	[M + H]^+^
54	Hesperidin	7.63	C_28_H_34_O_15_	609.1827	609.1825	0.33	[M − H]^−^
55	Neohesperidin	7.86	C_28_H_34_O_15_	609.1830	609.1825	0.82	[M − H]^−^
56	Azelaic acid	7.94	C_9_H_16_O_4_	187.0984	187.0976	4.28	[M − H]^−^
57	Isoliquiritin apioside	8.31	C_26_H_30_O_13_	551.1779	551.1759	3.63	[M + H]^+^
58	Neoliquiritin	8.47	C_21_H_22_O_9_	417.1209	417.1191	4.32	[M − H]^−^
59	Neoisoliquiritin	8.65	C_21_H_22_O_9_	417.1211	417.1191	4.79	[M − H]^−^
60	Liquiritin apioside	8.73	C_26_H_30_O_13_	551.1782	551.1759	4.17	[M + H]^+^
61	Daidzein	8.76	C_15_H_10_O_4_	255.0663	255.0652	4.31	[M + H]^+^
62	Spinacetin	9.07	C_17_H_14_O_8_	347.0777	347.0761	4.61	[M + H]^+^
63	Clycosin	9.62	C_16_H_12_O_5_	283.0616	283.0611	1.77	[M − H]^−^
64	β-Rhamnocitrin	9.69	C_16_H_12_O_7_	317.0670	317.0656	4.42	[M + H]^+^
65	Licoricesaponin A3	9.91	C_48_H_72_O_21_	983.4540	983.4493	4.78	[M − H]^−^
66	Britannilactone	10.03	C_17_H_24_O_5_	309.1709	309.1696	4.20	[M + H]^+^
67	Carabrone	10.04	C_15_H_20_O_3_	249.1497	249.1485	4.82	[M + H]^+^
68	Glabrolide	10.13	C_30_H_44_O_4_	469.3330	469.3312	3.84	[M + H]^+^
69	Hexahydrocurcumin	10.25	C_21_H_26_O_6_	373.1675	373.1657	4.82	[M − H]^−^
70	1-O-Acetylbritannilatone	10.28	C_17_H_24_O_5_	309.1709	309.1696	4.20	[M + H]^+^
71	Tomentosin	10.93	C_15_H_20_O_3_	249.1497	249.1485	4.82	[M + H]^+^
72	Aromaticin	11.24	C_15_H_18_O_3_	247.1341	247.1329	4.86	[M + H]^+^
73	Licorice saponin G2	11.25	C_42_H_62_O_17_	839.4091	839.4060	3.69	[M + H]^+^
74	Licorice saponin E2	11.27	C_42_H_60_O_16_	819.3841	819.3809	3.91	[M − H]^−^
75	Isomer of Licorice saponin G2	11.77	C_42_H_62_O_17_	839.4092	839.4060	3.81	[M + H]^+^
76	Melliferone	11.82	C_30_H_44_O_3_	453.3379	453.3363	3.53	[M + H]^+^
77	Glycyrrhizic acid	11.84	C_42_H_62_O_16_	821.3987	821.3965	2.68	[M − H]^−^
78	Formononetin	11.95	C_16_H_12_O_4_	269.0819	269.0808	4.09	[M + H]^+^
79	11-Deoxoglycyrrhizin	12.28	C_42_H_64_O_15_	807.4183	807.4172	1.36	[M − H]^−^
80	Isomer of Melliferone	12.44	C_30_H_44_O_3_	453.3380	453.3363	3.75	[M + H]^+^
81	(18β,20α)-Glycyrrhizic acid	12.48	C_42_H_62_O_16_	821.3972	821.3965	0.85	[M − H]^−^
82	Ivangustin	12.53	C_15_H_20_O_3_	249.1496	249.1485	4.42	[M + H]^+^
83	Glycycoumarin	12.55	C_21_H_20_O_6_	369.1348	369.1333	4.06	[M + H]^+^
84	Licoricesaponin J2	13.00	C_42_H_64_O_16_	823.4136	823.4122	1.70	[M − H]^−^
85	Casticin	13.29	C_19_H_18_O_8_	375.1089	375.1074	4.00	[M + H]^+^
86	6-Shogaol	13.35	C_17_H_24_O_3_	277.1799	277.1798	0.36	[M + H]^+^
87	Isomer of 11-Deoxoglycyrrhizin	13.51	C_42_H_64_O_15_	807.4188	807.4172	1.98	[M − H]^−^
88	6-Gingerol	13.81	C_19_H_30_O_4_	293.1767	293.1758	3.07	[M − H]^−^
89	Licochalcone D	14.16	C_21_H_22_O_5_	355.1542	355.1540	0.56	[M + H]^+^
90	Licoisoflavanone	14.35	C_20_H_18_O_6_	355.1187	355.1176	3.10	[M + H]^+^
91	Licoflavonol	14.78	C_20_H_18_O_6_	355.1187	355.1176	3.10	[M + H]^+^
92	4-Epi-isoinuviscolide	14.84	C_15_H_20_O_3_	249.1496	249.1485	4.42	[M + H]^+^
93	Dibritannilactone B	14.97	C_34_H_46_O_9_	599.3233	599.3215	3.00	[M + H]^+^
94	Japonicones D	15.14	C_34_H_44_O_9_	597.3075	597.3058	2.85	[M + H]^+^
95	8-Shogaol	16.03	C_19_H_28_O_3_	305.2126	305.2111	4.91	[M + H]^+^
96	1β-Hydroxyalantolactone	16.06	C_15_H_20_O_3_	249.1492	249.1485	2.81	[M + H]^+^
97	18-β-Glycyrrhetinic acid	16.43	C_30_H_46_O_4_	471.3488	471.3469	4.03	[M + H]^+^
98	Glyasperin D	16.97	C_22_H_26_O_5_	371.1868	371.1853	4.04	[M + H]^+^
99	10-Shogaol	18.48	C_21_H_32_O_3_	333.2437	333.2424	3.90	[M + H]^+^
100	Oleanolic acid	21.71	C_30_H_48_O_3_	455.3556	455.3535	4.61	[M − H]^−^
101	Ursolic acid	22.04	C_30_H_48_O_3_	455.3554	455.3535	4.17	[M − H]^−^
102	Stearamide	24.23	C_18_H_37_NO	284.2961	284.2948	4.57	[M + H]^+^
103	Erucamide	26.49	C_22_H_43_NO	338.3433	338.3417	4.73	[M + H]^+^

**Table 3:** Analysis of the blood-absorbed components of XDD through HPLC

No	Identification	t_*R*_ (min)	Formula	Mass error (ppm)	Adducts
1	Codonopsine	2.97	C_14_H_21_NO_4_	4.48	[M + H]^+^
2	Liquiritin	6.79	C_21_H_22_O_9_	1.68	[M − H]^−^
3	Neoliquiritin	8.43	C_21_H_22_O_9_	4.79	[M − H]^−^
4	β-Rhamnocitrin	9.67	C_16_H_12_O_7_	4.73	[M + H]^+^
5	Britannilactone	10.02	C_17_H_24_O_5_	3.88	[M + H]^+^
6	Licorice saponin G2	11.25	C_42_H_62_O_17_	2.50	[M + H]^+^
7	Isomer of licorice saponin G2	11.81	C_42_H_62_O_17_	3.81	[M + H]^+^
8	Glycyrrhizic acid	11.85	C_42_H_62_O_16_	3.29	[M − H]^−^
9	Melliferone	11.86	C_30_H_44_O_3_	4.41	[M + H]^+^
10	11-Deoxoglycyrrhizin	12.28	C_42_H_64_O_15_	1.73	[M − H]^−^
11	(18β,20α)-Glycyrrhizic acid	12.46	C_42_H_62_O_16_	3.77	[M − H]^−^
12	Isomer of melliferone	12.51	C_30_H_44_O_3_	3.75	[M + H]^+^
13	6-Gingerol	13.81	C_19_H_30_O_4_	3.07	[M − H]^−^

**Fig. 9:**
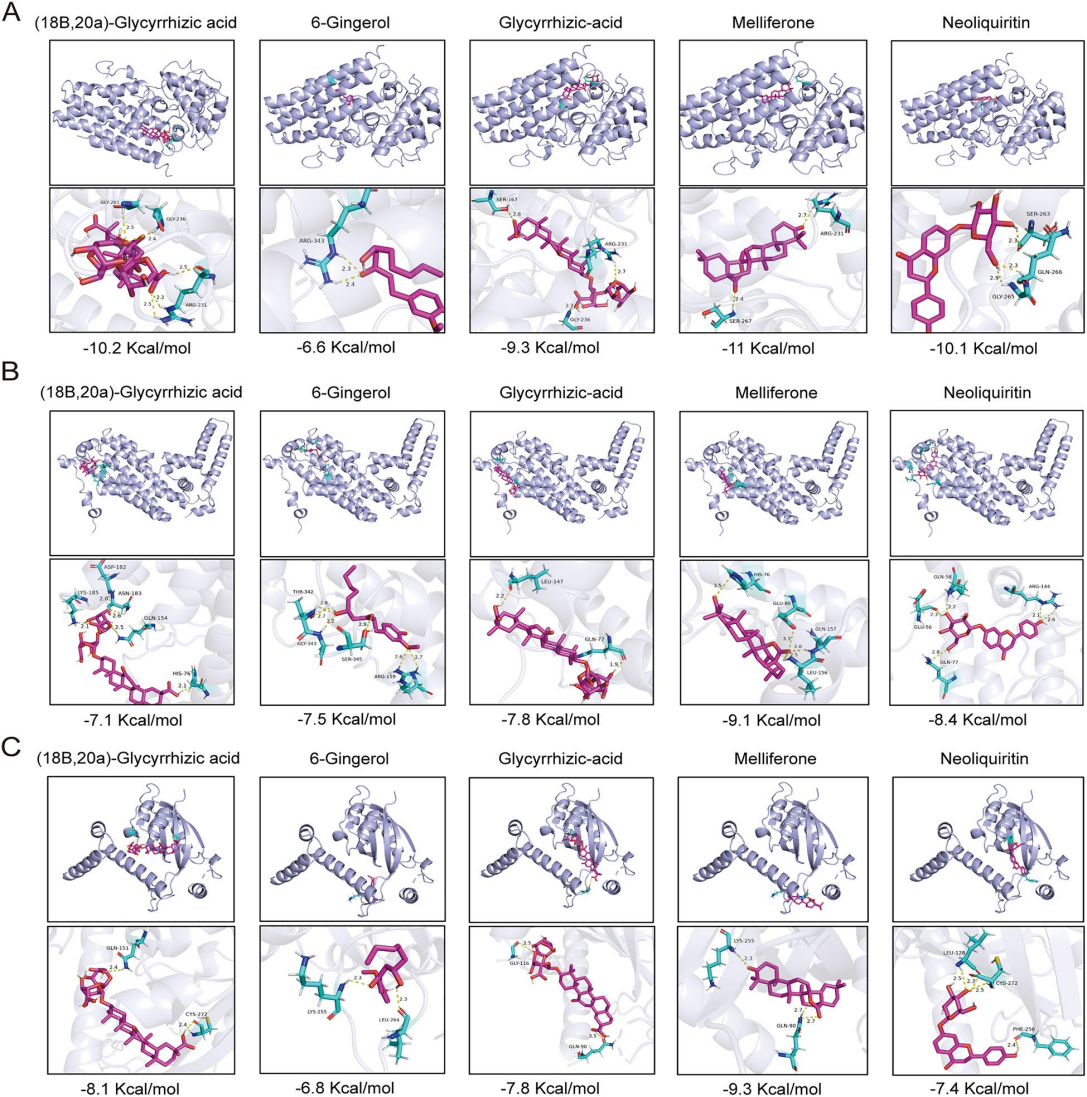
Molecular docking results of the top five bioactive components from XDD with key targets. **A** IDO, **B** TDO, and **C** AHRR, respectively

To investigate the potential mechanism of XDD in the treatment of RE, the top five potential active components identified from plasma pharmacokinetic analysis (based on peak area) were selected for molecular docking with key targets in the tryptophan-KYN-AhR-Th17 axis, namely IDO1, TDO2, and AHRR (Fig. 9A–C).

The results demonstrated that all five blood-absorbed components exhibited favorable binding affinity with the aforementioned targets. Among them, Melliferone showed the strongest binding to AHRR, with a binding energy of − 9.3 kcal/mol. Its binding mode was similar to that of the native ligand, stably occupying the ligand-binding pocket of AHRR. In addition, Neoliquiritin also displayed considerable inhibitory potential against both IDO and TDO, with binding energies of − 10.1 and − 8.4 kcal/mol, respectively, suggesting its possible role in blocking kynurenine production. These findings provide direct molecular evidence supporting the mechanism by which XDD interferes with the tryptophan-KYN- AhR signaling pathway and subsequently suppresses Th17-mediated inflammation.

**Fig. 10:**
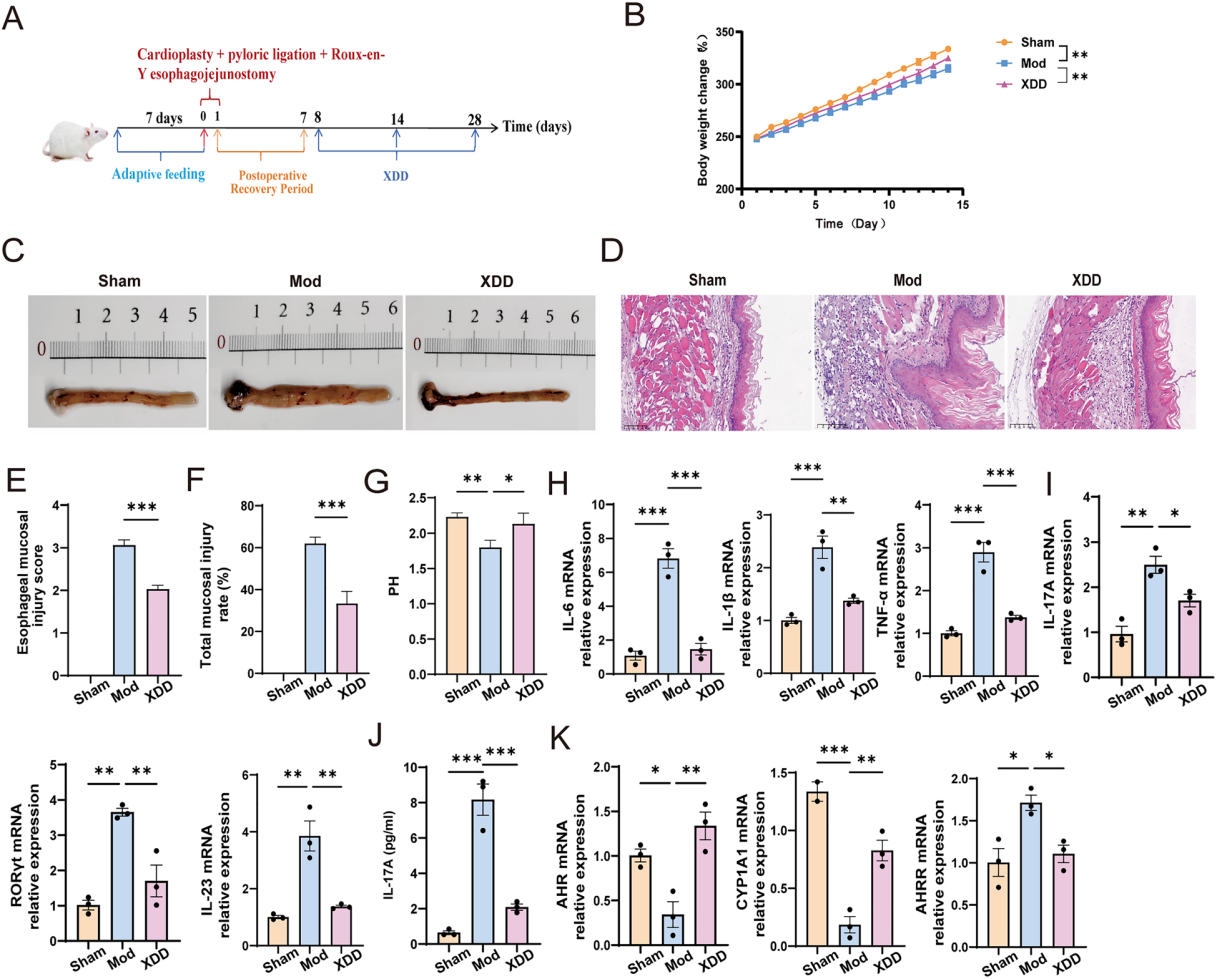
XDD Alleviates RE by Activating AhR and Inhibiting Th17 response. **A** Schematic diagram of RE rat model treated with Xuanfu Daizhe Decoction (XDD) (*n* = 6 per group). **B** Body weight changes during the experimental period. **C** The extent of esophageal injury in each group. **D** H&E-stained sections of esophageal tissues. **E** Esophageal mucosal injury scores. **F** Total mucosal injury rate. **G** The esophageal PH levels in different groups. **H** mRNA expression of pro-inflammatory cytokines (IL‑6, IL‑1β, TNF‑α) in esophageal tissues. **I** mRNA levels of IL-17A, RORγt and IL-23 in esophageal tissues. **J** Serum IL‑17A levels measured by ELISA. **K** mRNA expression of AhR pathway genes (AHR, CYP1A1, AHRR) in esophageal tissues. All data are presented as the mean ± SEMs of three independent experiments. Statistical significance was determined using one-way ANOVA with Tukey's post hoc test. *P < 0.05, **P < 0.01, ***P < 0.001

Having established the critical role of the KYN-AhR-AHRR axis in RE pathogenesis, we next evaluated the therapeutic potential of XDD in a rat RE model (Fig. 10A). The results showed that the body weight of rats was significantly decreased in the model group compared to Sham group; whereas XDD treatment significantly improved body weight loss (Fig. 10B). Histological analysis of esophageal tissues revealed that XDD markedly alleviated mucosal injury, inflammatory cell infiltration, and edema compared to the untreated model group (Fig. 10C, D). This was corroborated by significantly lower injury scores and injury rates (Fig. 10E, F). XDD also increased esophageal pH (Fig. 10G), indicating a reduction in pathological acidity, which is typically associated with RE.

We further analyzed the changes in inflammatory-related factors and the KYN signaling pathway. The results showed that XDD treatment significantly suppressed the mRNA expression of key inflammatory mediators (IL-6, IL-1β, TNF-α) (Fig. 10H) and Th17-associated factors (IL-17A, RORγt, IL-23) (Fig. 10I, J), while concurrently upregulating AhR and its target gene CYP1A1, and downregulating AHRR (Fig. 10K).

These in vivo results demonstrate that XDD effectively attenuates RE, restores AhR signaling and inhibits Th17-mediated inflammation.

### XDD decreases epithelial cell-derived kynurenine to attenuate Th17 response

**Fig. 11:**
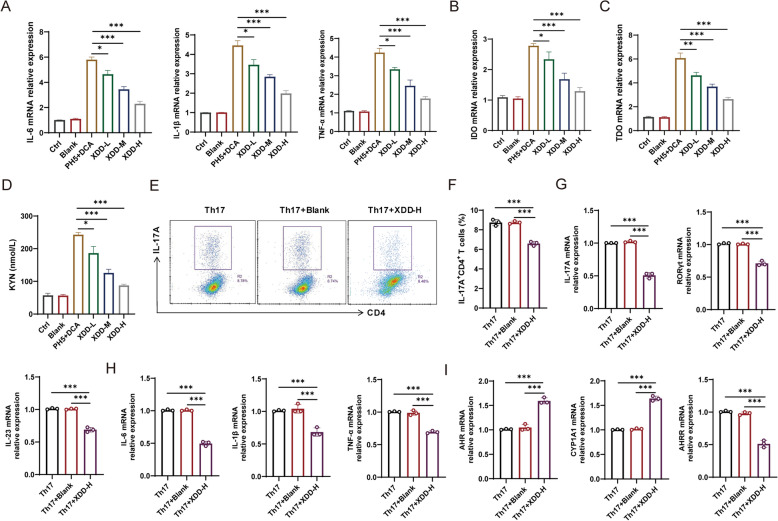
XDD decreases epithelial cell-derived kynurenine to attenuate Th17 activation. **A** mRNA expression of IL-6, IL-1β, and TNF-α in HEECs treated PH5 + DCA following intervention with low-, medium-, and high- dose of XDD-containing serum. **B, C** mRNA levels of IDO1 (**B**) and TDO (**C**) in inflamed HEECs after treatment with low-, medium-, and high- dose of XDD-containing serum. **D** KYN concentration in cell supernatant measured by ELISA. **E, F** CD4^+^ T cells were differentiated into Th17 cells in the presence or absence of high-dose XDD -containing serum. Intracellular IL-17A expression was analyzed by flow cytometry on day 5. **G–I** mRNA levels of Th17-related genes (IL‑17A, RORγt, IL‑23, IL‑6, IL‑1β, TNF‑α) and AhR pathway components (AHR, CYP1A1, AHRR) in Th17 cells (*n* = 3). All data are presented as the mean ± SEMs of three independent experiments. Statistical significance was determined using one-way ANOVA with Tukey's post hoc test. *P < 0.05, **P < 0.01, ***P < 0.001

To elucidate the cellular and molecular mechanisms underlying the therapeutic effects of XDD on RE, we employed an established in vitro inflammatory model using HEECs. Inflamed HEECs were treated with a fixed 10% (v/v) concentration of serum obtained from rats administered low-, medium-, or high-dose XDD (designated as XDD-L, XDD-M, and XDD-H serum, respectively). The medium dose was calculated as the clinically equivalent dose in rats based on human–rat body surface area conversion, and the low and high doses were set at 0.5-fold and twofold of the medium dose, respectively, following the widely used 0.5—twofold dose gradient recommended in previous study [19]. The results revealed that, compared to (PH5 + DCA) induced inflamed HEEC group, treatment with XDD-containing serum dose-dependently reduced the mRNA expression of inflammatory cytokines (IL-6, IL-1β, TNF-α) (Fig. 11A), and the key kynurenine-pathway enzymes IDO1 and TDO (Fig. 11B, C), leading to a corresponding decrease in KYN production (Fig. 11D).

Moreover, conditioned medium from HEECs treated with high-dose XDD serum (XDD-H) decreased the capacity to enhance IL-17A production in Th17 cells, as shown by flow cytometry (Fig. 11E, F). PCR analysis confirmed that XDD treatment reversed the inflammatory factors expression in Th17 cells, downregulating IL-17A, RORγt, and related cytokines (IL-23, IL-6, IL-1β and TNF-α), while restoring a balanced AhR pathway profile: increased AHR, CYP1A1; decreased AHRR (Fig. 11G–I). These data indicate that XDD exerts its therapeutic effect by directly acting on esophageal epithelial cells to normalize kynurenine metabolism, thereby breaking AHRR-dependent suppression of AhR signaling in Th17 cells.

## Discussion

This study provides the first systematic elucidation of the central role of the tryptophan- KYN metabolic axis in the pathogenesis of RE. By integrating clinical metabolomics, animal models, and in vitro cellular experiments, we demonstrated significantly elevated KYN levels in the serum of RE patients and experimental animals, and identified inflamed HEECs as a major source of KYN. More importantly, we highlighted a immunometabolic regulatory pathway whereby KYN disrupts the signaling balance between the AhR and its repressor AHRR, thereby driving Th17 cell differentiation and proliferation and exacerbating esophageal inflammatory injury. Furthermore, for the first time from the immunometabolic perspective, we revealed the regulatory mechanism of classic Chinese herbal formula XDD in the treatment of RE.

Tryptophan metabolism is essential for maintaining immune and metabolic homeostasis. Under physiological conditions, the conversion of tryptophan to KYN via the rate-limiting enzymes IDO1 and tryptophan 2,3-dioxygenase (TDO) ensures balanced immune tolerance. However, under pathological conditions such as RE, chronic inflammation markedly enhances IDO1/TDO expression, resulting in excessive KYN accumulation. creates a vicious cycle of immune dysregulation, in which excessive KYN acts not only as a tolerogenic signal but also as an inflammatory amplifier [27, 28]. Our results demonstrated significantly elevated KYN levels in both RE patients and animal models, consistent with observations in cancer and chronic inflammatory diseases [8], suggesting a shared metabolic mechanism underlying immune imbalance.

A pivotal finding of our study is the disrupted AhR-AHRR equilibrium in RE. AhR is a ligand-activated transcription factor that senses endogenous metabolites such as KYN [29]. Activation of AhR modulates both detoxification genes (e.g., CYP1A1) and immune cell differentiation [30, 31]. In our study, we found that KYN/AhR axis promoted Th17 differentiation and increased the expression of pro-inflammatory cytokines including IL-17A and IL-23, in line with prior studies in inflammatory bowel disease (IBD) [32, 33] and psoriasis [34, 35]. Interestingly, we observed significant upregulation of the AHRR in RE models. Its overexpression likely suppresses downstream AhR targets (e.g., CYP1A1), destabilizing the signaling balance and exacerbating inflammation [36]. This suggests that this AhR-AHRR imbalance represents a novel immunometabolic defect characteristic of RE pathogenesis.

Th17 cells are increasingly recognized as pivotal effectors in chronic inflammatory diseases. In RE, our flow cytometry and qPCR analyses demonstrated that KYN derived from HEECs strongly enhanced Th17-associated cytokines, particularly IL-17A and IL-23. These cytokines promote neutrophil recruitment and compromise the mucosal barrier, hallmarks of severe RE. The pathogenic role of the KYN-AhR-Th17 axis in RE shows remarkable parallels with its involvement in inflammatory bowel disease and psoriasis, underscoring its significance as a shared pathway in chronic inflammation [37, 38]. Importantly, the dysregulation of the Th17/Treg balance, driven by excessive KYN, favors Th17 differentiation while impairing Treg function, thereby perpetuating uncontrolled inflammation [39].

Our investigation provides the first systematic evidence demonstrating that XDD, originating from the classical *Shanghan Lun*, exerts its therapeutic effects in RE through immunometabolic regulation. Specifically, XDD downregulated IDO1/TDO expression, reducing KYN production and blocking its pro-inflammatory downstream effects. Moreover, XDD restored the AhR–CYP1A1 axis and alleviated the aberrant upregulation of AHRR, collectively mitigating Th17 polarization and esophageal inflammation. Thus, XDD functions not merely as a symptomatic gastrointestinal remedy but as a regulator of upstream metabolic-immune interactions.

A notable finding of our study is the dose-dependent efficacy of XDD. With increasing concentrations of XDD-containing serum, inhibition of HEEC-derived KYN production became more pronounced, and suppression of Th17 proliferation and IL-17A secretion was significantly enhanced. This suggests two important implications: (i) in clinical practice, dosage optimization of XDD could be critical to achieve maximal therapeutic efficacy, and (ii) standardization of dosage and preparation protocols is essential for ensuring consistent therapeutic outcomes in modernized TCM. These insights may guide both clinical decision-making and pharmaceutical quality control of XDD-based formulations.

Unlike conventional immunosuppressants that simply suppress immune effector cells, XDD exerts a dual role in immunometabolic regulation. On the one hand, it directly inhibits IDO1/TDO activity to reduce KYN accumulation; on the other, it modulates AhR signaling to restore Th17/Treg balance and re-establish immune homeostasis. This balanced mode of regulation avoids the pitfalls of excessive immunosuppression and instead provides a comprehensive approach to managing chronic inflammation. Such features position XDD as a promising candidate for treating not only RE but also a spectrum of immune-related inflammatory disorders.

While this study elucidates a novel mechanism in RE pathogenesis and the therapeutic potential of XDD, several limitations should be considered. First, our surgically-induced rat RE model, though mature and widely used, and effectively replicates the core immune-metabolic dysregulations (e.g., elevated KYN, enhanced Th17 response), it cannot fully replicate the entire spectrum of human RE, such as spontaneous onset or host-microbiome interactions. Second, our optimized in vitro model, while designed to approximate key features of human pathological reflux, employs a simplified, continuous exposure to a single bile acid. It does not replicate the intermittent nature of physiological reflux or the complex mixture of gastric acid, pepsin, and bile acids found in vivo. While recent studies have demonstrated that acidic bile salts can induce esophageal epithelial barrier dysfunction and inflammatory signaling [40], future studies incorporating multi-component refluxate models or advanced organotypic culture systems would provide additional pathophysiological relevance. Third, we did not examine XDD’s direct effects on isolated Th17 cells, which will be addressed in future studies. Fourth, other significantly altered metabolic pathways (e.g., linoleic acid metabolism) identified by metabolomics were not functionally validated, though they may regulate Th17 function and merit future exploration. Fifth, as RE is a chronic disease, the present study was based on short-term animal intervention and did not investigate the long-term efficacy, chronic toxicity, or potential adverse reactions of XDD under repeated administration. However, the herbal components of XDD have been widely used in clinical practice for decades with well-documented safety profiles. Recent clinical studies of the complete XDD formula have demonstrated its favorable safety in GERD/RE patients during 4-week to 4-month treatment courses, with no serious adverse reactions reported [41–43]. Future studies should incorporate long-term repeated-dose toxicity assessments and chronic efficacy observations to fully evaluate the safety profile of XDD in extended use. Sixth, we did not compare the therapeutic efficacy of XDD with first-line clinical treatments for RE such as PPIs, making it difficult to assess its relative advantages and precise clinical positioning. Additionally, further research is needed to explore the individual roles of XDD’s potential active components in regulating the KYN/AhR/Th17 axis, and investigate its long-term efficacy and safety in RE and other Th17-related disorders.

In conclusion, our work establishes aberrant tryptophan-KYN metabolism as a signature of RE immunopathology, unveils AHRR as a key regulator in this process, and provides a mechanistic basis for the application of XDD as a promising immunometabolic therapy for RE and potentially other chronic inflammatory diseases.

## Conclusion

**Fig. 12:**
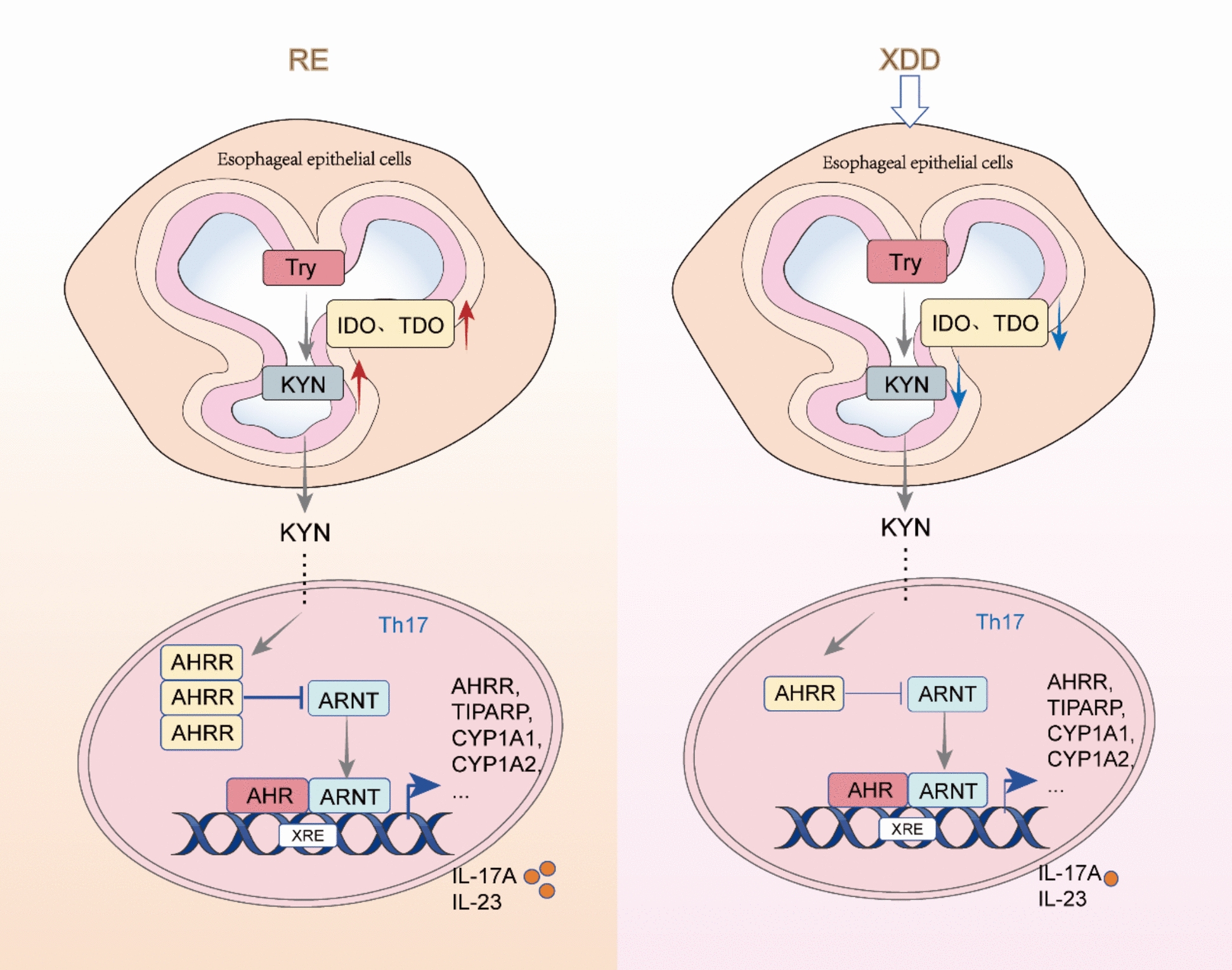
Proposed mechanism by which esophageal epithelial cell-derived KYN drives Th17 Inflammation in RE and therapeutic targeting by XDD. Schematic summary illustrating how inflamed HEECs upregulate IDO1/TDO, leading to KYN accumulation. KYN promotes Th17 cell differentiation and cytokine production via dysregulation of the AhR–AHRR–CYP1A1 axis. XDD alleviates esophageal inflammation by suppressing KYN production and restoring AhR signaling, thereby inhibiting Th17-mediated immunopathology

In summary, our work elucidates the tryptophan–KYN–AhR–Th17 axis as a pivotal pathogenic driver in RE. We provide mechanistic evidence that XDD alleviates disease pathology by simultaneously reducing KYN bioavailability and rebalancing AhR/AHRR signaling, thereby suppressing Th17-mediated inflammation (Fig. 12). These findings expand the mechanistic understanding of TCM in immune–metabolic regulation and support the therapeutic potential of XDD in RE.

## Supplementary Information

**Additional file 1. Table S1. **Primer sequences for real-time qPCR

## Data Availability

All data gathered during this study can be accessed from the corresponding authors upon request.

## Abbreviations

RE: Reflux esophagitis
Th17: T helper 17
XDD: Xuanfu Daizhe decoction
TCM: Traditional Chinese Medicine
HEECs: Human esophageal epithelial cells
KYN: Kynurenine
AHRR: Aryl hydrocarbon receptor repressor
AHR: Aryl hydrocarbon receptor
IL-17A: Interleukin-17A
IDO1: Indoleamine 2,3-dioxygenase 1
Treg: Regulatory T
Foxp3: Forkhead box P3
HCs: Healthy controls
LA: The Los Angeles
BMI: Body mass index
UPLC-QTOF-MS: Liquid chromatography-mass spectrometry
UHPLC–MS/MS: Ultrahigh-performance liquid chromatography-tandem mass spectrometry
RT‒qPCR: Reverse transcription quantitative real-time PCR
HPLC: High-performance liquid chromatography
QqQ MS: Quadrupole mass spectrometer
MRM: Multiple reaction monitoring
PBMCs: Peripheral blood mononuclear cells
FBS: Fetal bovine serum
DCA: Deoxycholic acid
CCK-8: Cell counting Kit-8
cDNA: Complementary DNA
qRT-PCR: Quantitative real-time PCR
GSRS: Gastroesophageal Reflux Disease Symptom Scale
RDQ: Reflux Disease Questionnaire
TDO: Tryptophan 2,3-dioxygenase
IBD: Inflammatory bowel disease
