# Supplementary material for: Esophageal epithelial cell-derived kynurenine drives Th17 inflammation in reflux esophagitis and is targeted by Xuanfu Daizhe decoction
Source: Chin Med. 2026 Jul 15;21:192. doi: 10.1186/s13020-026-01413-0 (PMC13371025; doi:10.1186/s13020-026-01413-0)
Supplement: Supplementary file 1 — Additional file 1. Table S1. Primer sequences for real-time qPCR [file 13020_2026_1413_MOESM1_ESM.docx]

| **Table S1. Primer sequences for real-time qPCR**   \| Primers \| 5`-3` \| \| --- \| --- \| \| in humans \| \| \| AHR \| F: AATCCAGTACTGCCAGGCCAAC \| \| R: GGTCTGGCTTCTGACGGATGA \| \| AHRR \| F: CTGACCCGCTGCTTCATCTG \| \| R: ATCGTCATGAGTGGCTCGGG \| \| CYP1A1 \| F: TCCGGGACATCACAGACAGC \| \| R: ACCCTGGGGTTCATCACCAA \| \| IL-17A \| F: TCCCACGAAATCCAGGATGC \| \| R: GGATGTTCAGGTTGACCATCAC \| \| IL-23 \| F: GAGCCTTCTCTGCTCCCTGATA \| \| R: GACTGAGGCTTGGAATCTGCTG \| \| RORyt \| F: GTGGGGACAAGTCGTCTGG \| \| R: AGTGCTGGCATCGGTTTCG \| \| IDO1 \| F: CAGCTGCTTCTGCAATCAAA \| \| R: AGCGCCTTTAGCAAAGTGTC \| \| TDO \| F: CAAATCCTCTGGGAGTTGGA \| \| R: GTGCATCCGAGAAACAACCT \| \| IL-6 \| F: AGACAGCCACTCACCTCTTCAG \| \| R: TTCTGCCAGTGCCTCTTTGCTG \| \| IL-8 \| F: GAGAGTGATTGAGAGTGGACCAC \| \| R: CACAACCCTCTGCACCCAGTTT \| \| IL-1β \| F: CCACAGACCTTCCAGGAGAATG \| \| R: GTGCAGTTCAGTGATCGTACAGG \| \| TNF-α \| F: GAGGCCAAGCCCTGGTATG \| \| R: CGGGCCGATTGATCTCAGC \| \| GAPDH \| F: GAAGGTGAAGGTCGGAGT \| \| R: GAAGATGGTGATGGGATTTC \| \| in rats \| \| \| RORγt \| F: CCATTGTTTCCCTCCATCCCATCC \| \| R: GTTAGCACAGCACAGACCTCACTC \| \| CYP1A1 \| F: TGGAGCCTCATGTACCTGGTAACC \| \| R: CGATCCCTGCCAATCACTGTGTC \| \| IL-17A \| F: CTTCTGTGATCTGGGAGGCA \| \| R: GGCGGACAATAGAGGAAACG \| \| IL-23 \| F: CTCAGGGACAACAGTCAGTTC \| \| R: ACAGGGCTATCAGGGAGCA \| \| AHR \| F: GGATGAAGAAGGACGCGAAC \| \| R: TCCTTACTCGGGGTTGACTG \| \| AHRR \| F: TTCATTTGTCGTGTCCGCTG \| \| R: CGCCACAATGCAAAACAAGG \| \| GAPDH \| F: AGTCTACTGGCGTCTTCACC \| \| R: CCACGATGCCAAAGTTGTCA \| \| IDO1 \| F: TGGGCTTTGCTCTACCACAT \| \| R: TGTGTCCCCTCAGTTCTTCG \| \| TDO \| F: TTCCGGAAGCAGAAAGAGGT \| \| R: GGAAAGGGACCTGGAATCGA \| \| IL-6 \| F: CCACCCACAACAGACCAGTA \| \| R: ACTCCAGAAGACCAGAGCAG \| \| IL-8 \| F: TCATCCCAGGAGCAAAGAGG \| \| R: ACCGTGTCAATCCTCCAAGT \| \| IL-1β \| F: CTCTGTGACTCGTGGGATGA \| \| R: AGATTCTTCCCCTTGAGGCC \| \| TNF-α \| F: GCTCCCTCTCATCAGTTCCA \| \| R: AGCCTTGTCCCTTGAAGAGA \|      \| \| \| \|  \| \| --- \| \| \| --- \| --- \| \| \| --- \| --- \| --- \| \| \| --- \| --- \| --- \| --- \| |
| --- | --- | --- | --- | --- | --- | --- | --- | --- | --- | --- | --- | --- | --- | --- | --- | --- | --- | --- | --- | --- | --- | --- | --- | --- | --- | --- | --- | --- | --- | --- | --- | --- | --- | --- | --- | --- | --- | --- | --- | --- | --- | --- | --- | --- | --- | --- | --- | --- | --- | --- | --- | --- | --- | --- | --- | --- | --- | --- | --- | --- | --- | --- | --- | --- | --- | --- | --- | --- | --- | --- | --- | --- | --- | --- | --- | --- | --- | --- | --- | --- | --- | --- | --- | --- | --- | --- | --- | --- |
